# The Speciation and Coordination of a Deep Earth Carbonate‐Silicate‐Metal Melt

**DOI:** 10.1029/2021JB023314

**Published:** 2022-03-20

**Authors:** A. H. Davis, N. V. Solomatova, A. J. Campbell, R. Caracas

**Affiliations:** ^1^ Department of the Geophysical Sciences University of Chicago Chicago IL USA; ^2^ CNRS Ecole Normale Supérieure de Lyon Laboratoire de Géologie de Lyon LGLTPE UMR5276 Centre Blaise Pascal Lyon France; ^3^ The Center for Earth Evolution and Dynamics (CEED) University of Oslo Oslo Norway

## Abstract

Ab initio molecular dynamics calculations on a carbonate‐silicate‐metal melt were performed to study speciation and coordination changes as a function of pressure and temperature. We examine in detail the bond abundances of specific element pairs and the distribution of coordination environments over conditions spanning Earth’s present‐day mantle. Average coordination numbers increase continuously from 4 to 8 for Fe and Mg, from 4 to 6 for Si, and from 2 to 4 for C from 1 to 148 GPa (4,000 K). Speciation across all pressure and temperature conditions is complex due to the unusual bonding of carbon. With the increasing pressure, C‐C and C‐Fe bonding increase significantly, resulting in the formation of carbon polymers, C‐Fe clusters, and the loss of carbonate groups. The increased bonding of carbon with elements other than oxygen indicates that carbon begins to replace oxygen as an anion in the melt network. We evaluate our results in the context of diamond formation and of metal‐silicate partitioning behavior of carbon. Our work has implications for properties of carbon and metal‐bearing silicate melts, such as viscosity, electrical conductivity, and reactivity with surrounding phases.

## Key Points

- At high pressure, carbon begins to replace oxygen instead of silicon in carbonate‐silicate‐metal liquid networks
- C‐C and C‐Fe bond abundances increase with pressure at the expense of C‐O bonding
- Carbonate‐silicate‐metal melts may be parent melts for diamonds or dense C‐Fe melts in the lower mantle

## 1 Introduction

Carbon is ubiquitous in the Earth’s crust, but the amount and role of carbon in the deep Earth is less well understood. Carbon is regularly subducted into the mantle in the form of carbonates (Kelemen & Manning, 2015; Tao et al., 2018). However, estimates of the amount of carbonate that survives into the lower mantle vary from 0.0001 to 52 Mt per year (Dasgupta & Hirschmann, 2010; Kelemen & Manning, 2015). The large discrepancy in estimation stems from carbon’s varied behavior under specific thermodynamic and chemical conditions. For instance, carbonates are known to melt at relatively low temperatures (at 21 GPa, ∼2,000 K for CaCO_3_ and ∼1,350 K for carbonated MORB; Li et al., 2017; Thomson et al., 2016), to form diamonds and carbides under reducing conditions (Rohrbach & Schmidt, 2011; Stachel & Luth, 2015), and to react with surrounding mantle phases (Dorfman et al., 2018; Lv et al., 2021). However, recent evidence indicates that carbonates could be present in the lower mantle. Inclusions in deep Earth diamonds contain carbonates coexisting with lower mantle phases (Agrosì et al., 2019; Korsakov & Hermann, 2006). Recent measurements of the metal‐silicate partitioning coefficient of carbon show that carbon is less siderophile than originally thought (Fichtner et al., 2021; Fischer et al., 2020), indicating that less carbon is sequestered in the core, and could be stored in the mantle instead.

If carbonates are stable and present in the lower mantle, they are likely reacting with surrounding mantle phases. Previous studies of carbonate reactions in the lower mantle focus on reactions of solid carbonates with silicates and metals (Dorfman et al., 2018; Lv et al., 2021; Martirosyan et al., 2016). However, few studies have examined carbonate melt interactions in the lower mantle. Carbonates have relatively low melting temperatures, allowing for generation of melt at present day mantle conditions. Additionally, the presence of volatiles such as carbon or water is known to cause melting point depression in silicates (Dasgupta et al., 2007). Understanding the behavior and interaction of carbonate melts in the Earth’s interior is important to understand Earth’s formation and evolution. In the early Earth, carbonate melts could react with both silicate and metallic melts in the magma ocean, influencing how carbon was sequestered and stored. In the present day, carbonate, silicate, and metal melts could interact and mix freely at the core‐mantle boundary, influencing the composition of the core and the density of melts in the deep mantle.

We examine the speciation and the coordination of a carbonate‐silicate‐metal melt at conditions relevant to a magma ocean and to the present‐day Earth’s lower mantle. Previous studies examine carbonate melts (Koura et al., 1996; Li et al., 2017; Xu et al., 2020), carbon‐bearing silicate melts (Bajgain & Mookherjee, 2021; Ghosh & Karki, 2017; Ghosh et al., 2017; Karki et al., 2020), carbon and iron‐bearing silicate melts (Karki et al., 2020; Solomatova & Caracas, 2021; Solomatova et al., 2019), and carbon partitioning between silicate and iron melts (Zhang & Yin, 2012). The melt composition of this study differs from that of previous studies in that our melt has subequal amounts of carbonate, silicate, and metal, and acts as a representative composition of the types of mixed melts that could be present in the deep Earth. We examine how coordination distribution, speciation, cation diffusivity, and electrical conductivity evolve as a function of pressure and temperature. These features have implications for melt properties, such as viscosity, miscibility, and reactivity with other mantle phases.

## 2 Methods

*Ab initio* molecular dynamics calculations were performed with density functional theory using the Vienna *ab initio* simulation package (Kresse & Furthmuller, 1996). The projector‐augmented wave method (Blochl, 1994) was used to represent the core electrons. The generalized gradient approximation in the Perdew–Burke–Ernzerhof form (Perdew et al., 1996) was used to treat electron exchange and correlation. The calculations were spin polarized, meaning that the d electron spin states were unconstrained at all temperatures and pressures. A Hubbard *U*_*eff*_ (U‐J) parameter of 4 eV was applied. In previous tests on iron‐bearing pyrolite melt, a *U*_*eff*_ value of 4 eV was found to increase the magnetic moment of the iron atoms with only a small pressure effect (<1 GPa; Caracas et al., 2019). The kinetic energy cutoffs for the plane‐wave expansion of the wavefunctions were set to 600 eV. We used the canonical ensemble with constant volume, temperature, and number of atoms and with a Nosé‐Hoover thermostat (Hoover, 1985; Nosé, 1984). Simulations were performed at 3,000 and 4,000 K and at volumes corresponding to pressures of 0–45 GPa at 3,000 K and 1–148 GPa at 4,000 K, with time steps of 1–2 fs for 18–80 ps. These conditions span the mantle in both pressure and temperature. The 3,000 K results are pertinent to melting at low pressures while the 4,000 K results are pertinent to melting at high pressures, particularly in this carbon‐rich system as the presence of volatiles is known to cause melting point depression in silicates (Ghosh et al., 2009). Additionally, performing calculations at both 3,000 and 4,000 K allows one to evaluate the effect of pressure on the melt in isolation from temperature effects, and vice versa.

To generate the starting melt configurations, a carbon‐bearing enstatite crystal was first created by substituting half of the silicon atoms with carbon atoms and then slowly heating it from 0 to 4,000 K at 0 GPa to make a carbonate‐silicate melt. This configuration was then brought to a target volume and 3,000 K. Iron atoms were then randomly distributed into voids in the carbonate‐silicate melt and short simulations of 200–500 fs were run to equilibrate the melt mixture and obtain starting configurations for the 3,000 and 4,000 K simulations. Additional configurations for each volume at both temperatures were obtained by taking the starting configuration from a nearby volume and either compressing or expanding the melt to its new target volume. All calculations were run with a minimum of two starting configurations, and results were averaged.

To ensure that simulations were molten at all volumes, the mean‐square displacements of the atoms were examined (see Figure S1, Supporting Information S1). Simulations of melts have mean‐square displacements that increase linearly with time, while glassy simulations have mean‐square displacements that level off with the increasing time. If the mean‐square displacements of the atoms leveled off and were anomalously low (i.e., not equal to at least the length of the box), the simulation was not included in our analysis. Simulations at 3,000 K and at pressures above 45 GPa were not included due to the low mean‐square displacement of the atoms suggesting glass‐like rather than melt behavior, but all simulations at 4,000 K were included. The Brillouin zone was sampled at the gamma point. The mean‐square displacement as a function of time shows a ballistic regime below approximately 1,000 fs, after which, the atoms reach a diffusive regime. For the density of states calculations, a k‐point grid of either 8 × 8 × 8 or 6 × 6 × 6 was used depending on the density of the melt.

**Figure 1:**
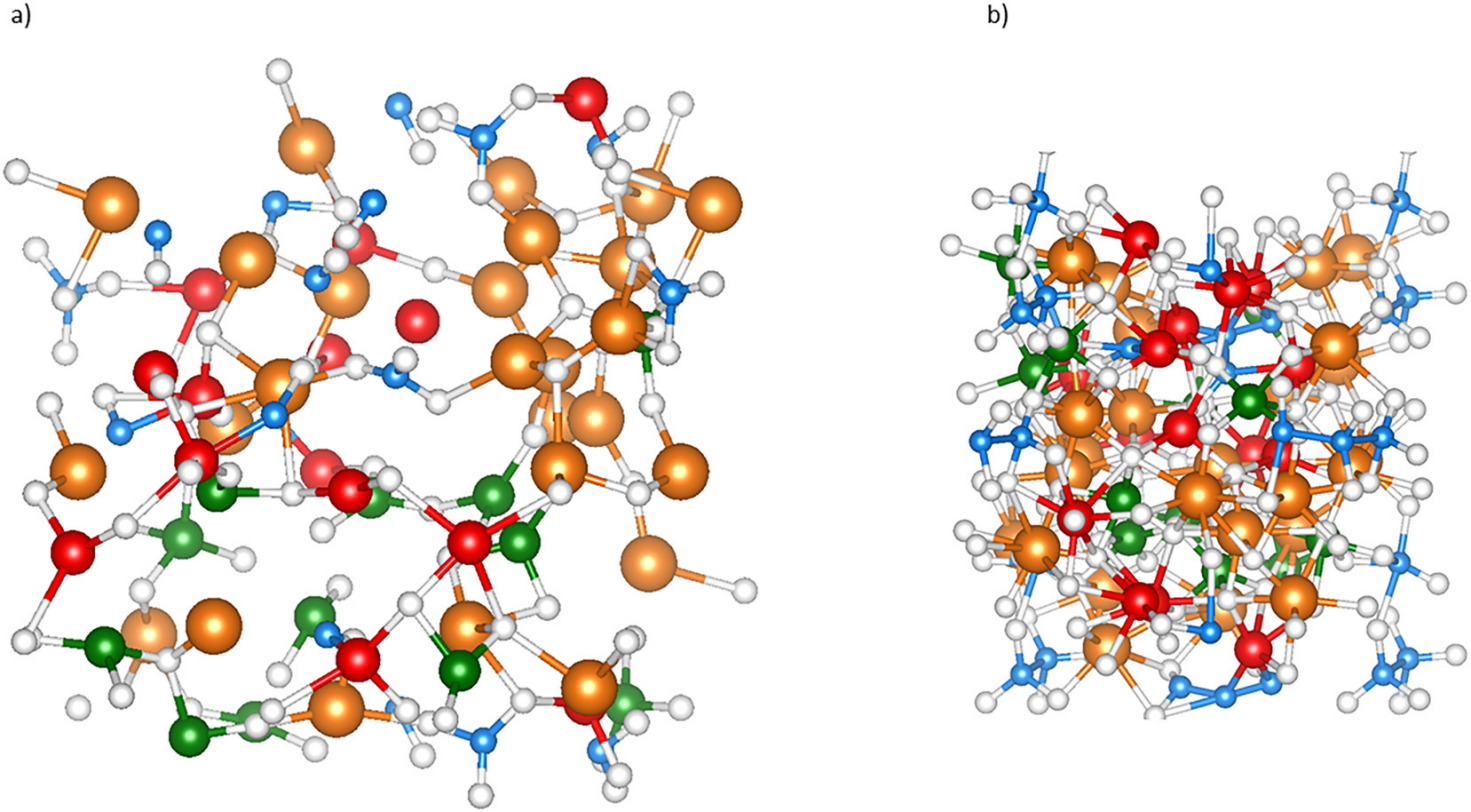
Structure of the carbonate‐silicate‐metal melt at (a) 1 GPa and 4,000 K and (b) 148 GPa and 4,000 K Carbon is blue, silicon is green, iron is red, magnesium is orange, and oxygen is white. At low pressure, carbon has one, two, or three‐fold coordination, and silicon has tetrahedral coordination. At high pressure, carbon adopts a tetrahedral coordination, and silicon is octahedrally coordinated.

The melt composition represents a carbonatite‐metal melt with stoichiometry Mg_24_Fe_13_Si_12_C_12_O_72_ (Figure 1). The composition can be split into 12 MgSiO_3_ units, 12 MgCO_3_ units, and 13 Fe units, for subequal amounts of silicate, carbonate, and iron metal. Additionally, a simulation of an oxidized melt with stoichiometry Mg_20_Fe_17_Si_12_C_12_O_72_ was conducted, where four magnesium atoms were replaced with four iron atoms to add ferrous rather than metallic iron to the starting composition. Bond distances were determined from the pair distribution functions (Figure S2, Supporting Information S1). The first peak in the pair distribution function approximates the average bond length, while the first minimum in the pair distribution function marks the radius of the first coordination sphere for the reference atom. This minimum translates to the maximum acceptable bond distance for a bonding pair, and the fitted minimum values were used in the speciation and coordination analysis.

## 3 Results and Discussion

Using *ab initio* molecular dynamics simulations on a carbonate‐silicate‐metal melt up to core‐mantle boundary pressures and temperatures of either 3,000 or 4,000 K, we evaluate the effects of pressure and temperature on interatomic bonds, chemical speciation, coordination states, cation diffusivity, and electrical conductivity.

### 3.1 Interatomic Bonds

**Figure 2:**
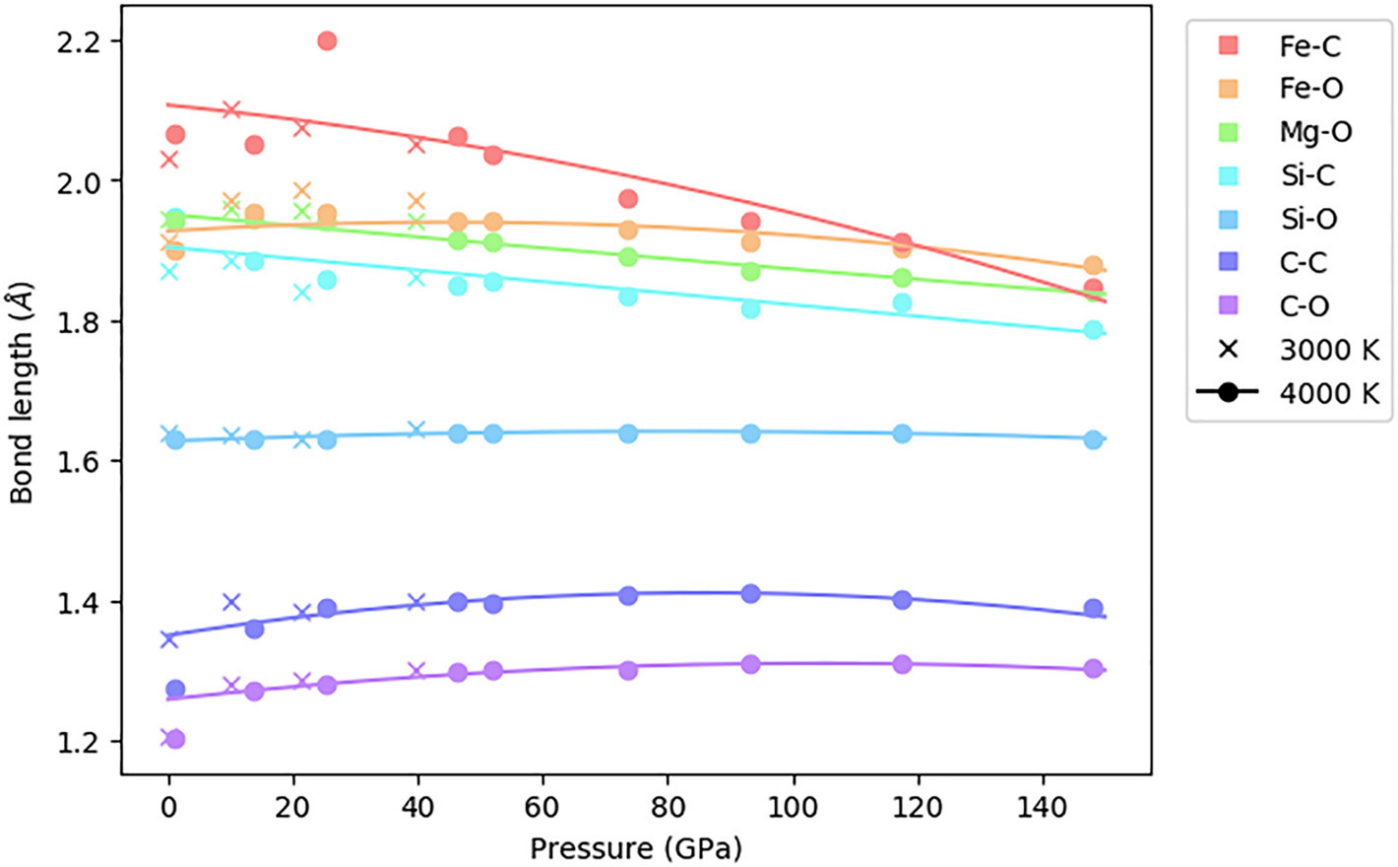
Average bond lengths of all bonding element pairs as a function of pressure, as estimated from the first maximum in the pair distribution function.

The most frequent bond distance for an element pair corresponds to the first maximum in the pair distribution function (Solomatova & Caracas, 2019; Figure S2, Supporting Information S1). We take this maximum to be the average bond length. At 3,000 K and ambient pressure, we find average bond distances of 1.21 Å for C‐O, 1.35 Å for C‐C, 1.64 Å for Si‐O, 1.87 Å for Si‐C, 1.91 Å for Fe‐O, 1.95 Å for Mg‐O, and 2.03 Å for C‐Fe (Figure 2). Between 3,000 and 4,000 K, bond lengths are similar, indicating that temperature does not have a large effect on bond length. Average bond length changes with depth as a function of the increasing pressure and increasing coordination. With no change in melt structure, bonds would shorten and polyhedra shrink as ions move closer together. However, with the increasing coordination, bonds can lengthen as polyhedra expand. At 4,000 K, C‐Fe, Si‐C, and Mg‐O bonds demonstrate the most constant decrease in length over the pressure range studied. Other bonds decrease in length less dramatically. For these bonds, the pressure effect is accommodated mostly in the second coordination sphere rather than the first. All bonds decrease in length beyond 117 GPa. However, at various pressure ranges below 117 GPa, Fe‐O, Mg‐O, Si‐O, C‐C, and C‐O bonds exhibit lengthening, corresponding to coordination changes in the cations (Figure 7a). Fe and Mg have similar coordination environments, and Mg‐O and Fe‐O bonds exhibit similar behavior, lengthening during a rapid increase in coordination from 1 to 20 GPa before decreasing as the coordination increase slows. Si‐O bonds lengthen between 15 and 70 GPa but remain largely unchanged over the entire pressure range studied. A rapid increase in silicon coordination between 0 and ∼70 GPa explains the lengthening of bonds between 15 and 70 GPa. In the first 15 GPa, pressure effects dominate the Si‐O bond length even as the coordination state of silicon increases. At 1 GPa, carbon species are molecular, and do not interact with the melt network. Thus, bond lengths at 1 GPa are far shorter than bond lengths at the other pressures studied. In fact, at 148 GPa, C‐O and C‐C bonds are longer than at 1 GPa (1.30 Å and 1.39 Å at 148 GPa, respectively, compared to 1.20 Å and 1.27 Å at 1 GPa). In the first 45 GPa, C‐O and C‐C bonds lengthen, corresponding to carbon’s threefold to fourfold coordination change, and begin to shorten beyond 45 GPa.

### 3.2 Chemical Speciation

**Figure 3:**
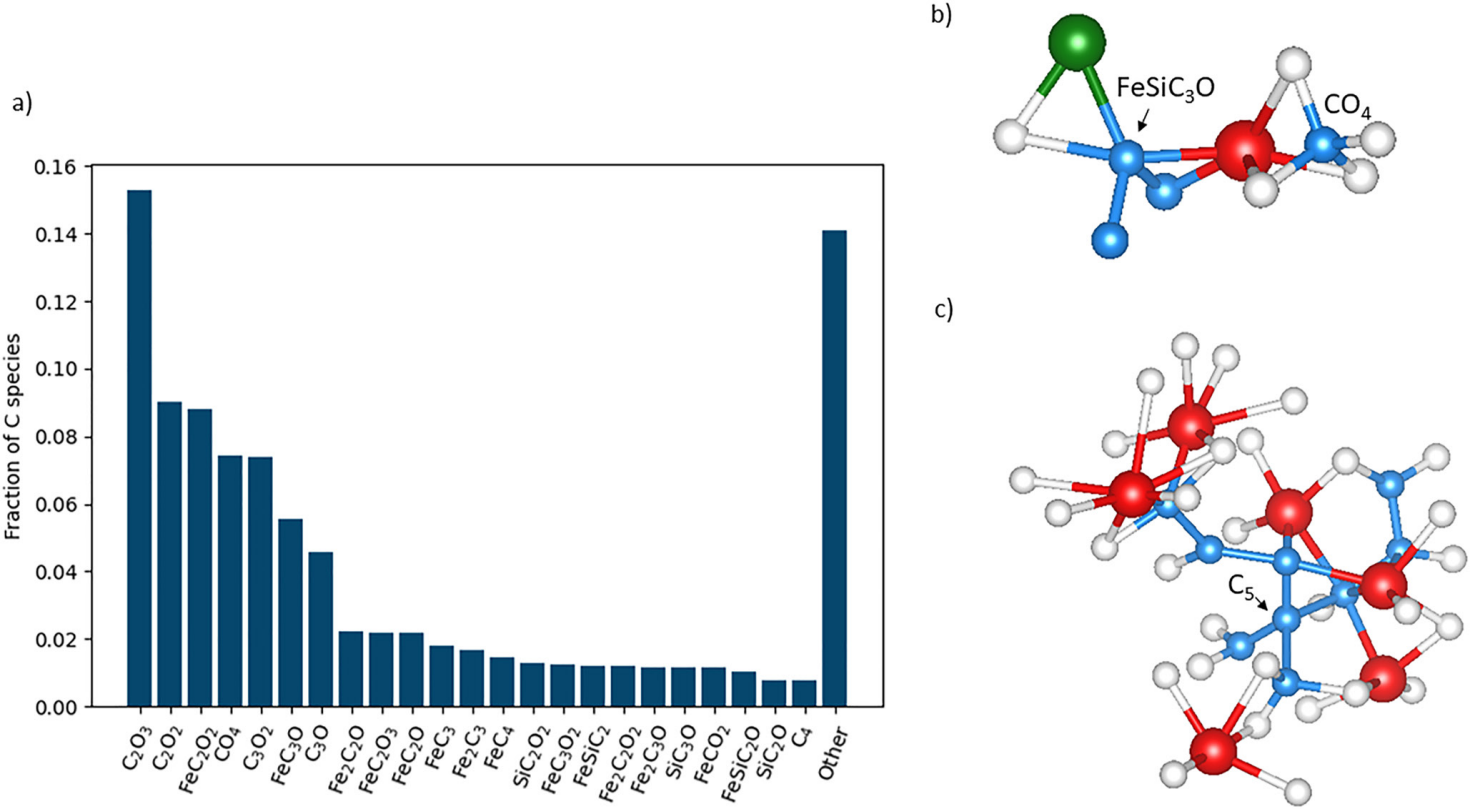
(a) Variety of carbon species formed at 117 GPa and 4,000 K and their relative abundances. Carbon forms a diversity of bonds, and many species are present. (b) and (c) Examples of carbon species. Atoms are carbon (blue), oxygen (white), silicon (green), and iron (red). Magnesium atoms have been omitted for clarity. (b) An example of a carbon atom exhibiting the four types of carbon bonds (C‐Fe, C‐C, C‐O, and C‐Si) (left) and an example of a carbonate group, where carbon is only bonded to oxygen (right). (c) An example of a carbon‐iron cluster consisting of nine carbon atoms and six iron atoms. A central carbon atom forms four C‐C bonds, and could be a seed nucleus for diamond formation.

Through the definition of interatomic bonds, we identify and quantify chemical species in the melt. In our analysis, we define a species as a central cation and the bonding atoms within the first coordination shell of that cation. Speciation in the simulated carbonate‐silicate‐metal melt is complicated due to carbon’s ability to act as both a cation and an anion. Carbon bonds to every element in the simulation except magnesium. Iron and silicon bond to both oxygen and carbon, and magnesium bonds only to oxygen. Carbon’s bonding versatility allows for the creation of a multitude of species (Figure 3a). Over the entire 46 ps simulation at 117 GPa and 4,000 K, C_2_O_3_, C_2_O_2_, and FeC_2_O_2_ account for 33% of carbon species, and another 171 species account for the remaining 67%. The species formed are quite diverse and often have unusual bonding environments. Some examples of different types of carbon bonding are displayed in Figures 3b and 3c. In Figure 3b, carbon forms direct bonds to carbon, oxygen, silicon, and iron simultaneously to create a FeSiC_3_O species. While FeSiC_3_O is an example of an unusual bonding environment, we see an example of a more conventional carbon group in close proximity, a carbonate (CO_4_), where carbon is bound only to oxygen. Figure 3c contains an example of a carbon‐iron cluster containing nine carbon atoms and six iron atoms, demonstrating carbon’s ability to polymerize and to bond with metal. Interestingly, the iron atoms are on the periphery of the carbon cluster, which contains a C_5_ species that could act as a seed nucleus for diamond formation. The arrangement of the iron and carbon atoms suggests that diamonds grow from metallic liquids in the mantle, as suggested previously through analysis of metallic inclusions in diamonds (Smith et al., 2016). Both images shown in Figure 3 are a sample of the varied species carbon forms.

**Figure 4:**
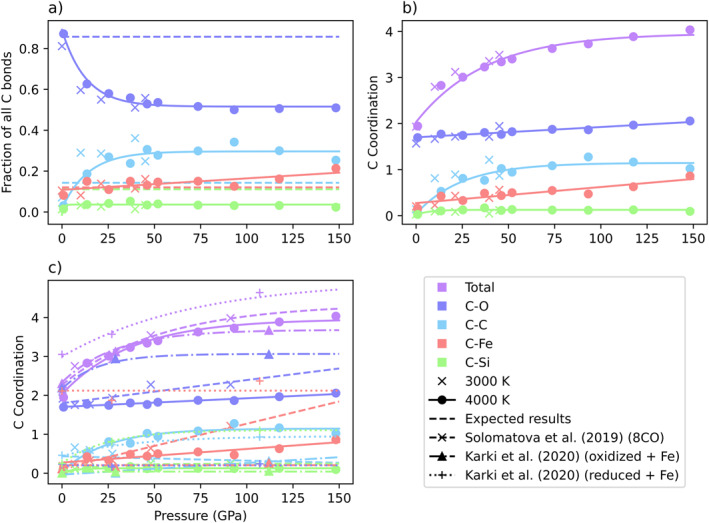
Carbon speciation as a function of pressure and temperature. (a) Displays the fraction of all carbon bonds in a simulation that are C‐O (purple), C‐C (blue), C‐Fe (red), and C‐Si (green) bonds. C‐C and C‐Fe bonding increase with pressure, while C‐O bonding decreases. Dashed lines are expected results from statistical sampling. Compared to statistical sampling, C‐C and C‐Fe bonding are more abundant than expected while C‐O and C‐Si bonding are less abundant than expected. (b) Displays the average coordination number of carbon to oxygen (purple), carbon (blue), iron (red), silicon (green), and to all elements (lilac). (c) Displays the average coordination of carbon as measured at 4,000 K in this study (solid lines, circles) versus Solomatova et al. (2019) (dashed lines, x’s) and Karki et al. (2020) (dashed dotted lines, triangles for oxidized melt and dotted lines, plusses for reduced melt).

To evaluate speciation changes as a function of pressure, we examine bond abundances. Carbon displays the most varied speciation of the cations studied. Figure 4a plots the fraction of all carbon bonds that are C‐X, where X is the bonding element to carbon, either O, C, Fe, or Si, as a function of pressure and temperature. The 1 GPa simulations have notably different C‐C and C‐O bond abundances compared to the other pressure points. C‐O bonds make up 87% of carbon bonds, while C‐C bonds are low, at only 3% of carbon bonds. This discrepancy arises because at 1 GPa, carbon forms mostly CO and CO_2_ molecules (Figure 7e). Beyond 1 GPa, carbon dissolves into the liquid and bonds more extensively with elements other than oxygen. In the 4,000 K results, C‐Fe and C‐C bonding both increase with pressure. C‐Fe bonding increases linearly. At 1 GPa, 8% of all carbon bonds are C‐Fe bonds, but at 148 GPa, 21% of all carbon bonds are C‐Fe bonds. C‐C bond abundances also increase with pressure, but the majority of the change happens within the first 25 GPa, from 3% at 1 GPa to 27% at 25 GPa. Subsequently, C‐O bonding shows an opposite trend with C‐C bonding, and decreases rapidly from 1 to 25 GPa. C‐O bond proportions fall from 87% at 1 GPa to 58% at 25 GPa to 51% at 148 GPa. C‐Si bond abundances rise from 1% at 1 GPa to 5% at 37 GPa, before falling back to 2% at 148 GPa. Speciation results at 3,000 and 4,000 K show similar trends in bond abundances. Figure 4b contains the same bonding information as Figure 4a, plotted in coordination space. As pressure increases, the total number of carbon bonds increases as the average coordination number rises (Figure 7). However, the relative proportions of individual bonds evolve with pressure, indicating carbon’s changing affinity for different elements. At 1 GPa and 4,000 K, carbon’s average coordination to oxygen is 1.7, which accounts for 87% of the total coordination. At 148 GPa and 4,000 K, carbon’s average coordination to oxygen is 2.1, which accounts for just 51% of the total coordination. The small rise in C‐O coordination is outpaced by more rapid rises in C‐C and C‐Fe coordination with pressure. From 1 to 148 GPa, C‐C coordination increases from 0.1 to 1.0, and carbon‐iron coordination increases from 0.2 to 0.9. Although carbon’s affinity for carbon and iron increases with pressure, at no point C‐C or C‐Fe bonding is more abundant than C‐O bonding. The overall effect is that as pressure increases, carbon begins to play a similar role as oxygen in the melt network, forming bonds to carbon, iron, and silicon. Carbon has been observed to substitute for oxygen in molten, amorphous, and crystalline silicate structures in previous NMR studies (Sen et al., 2013), indicating that carbon prefers to replace oxygen, an anion, instead of silicon, a cation, in the melt network.

To put these observations into context, we compare our 4,000 K simulation results to expected results from statistical sampling (Figure 4a). Statistical sampling is calculated by assuming that each cation has no chemical preference for any of the bonding atoms. In other words, what bond abundances would we expect to see if each cation coordinates with a randomized selection from the available anions? To do so, we identify the bonding atoms for each cation type and calculate the fraction of total bonds we expect for each cation‐anion pair. For example, we have identified that iron bonds only with carbon and oxygen. 84 anions (72 oxygen and 12 carbon) are available, and thus, 86% (72/84) of iron’s bonds should be to oxygen, and 14% (12/84) to carbon, assuming no chemical preference for either. For carbon, we expect the number of C‐C, C‐Fe, and C‐Si bonds to be similar due to the subequal amounts of the three elements. We also expect C‐O bonds to make up the majority of carbon bonds. At 1 GPa, C‐O bonding is more prevalent than expected, and C‐C, C‐Fe, and C‐Si bonding are all less prevalent. This result is unsurprising as carbon mostly forms molecular CO and CO_2_ at these conditions. However, above 1 GPa, C‐C and C‐Fe bonding are more prevalent than expected, while C‐O and C‐Si bonding are less prevalent. The discrepancy between expected and actual results only grows with the increasing pressure.

The large percentage of C‐C and C‐Fe bonds in our simulations suggests both a tendency for carbon to polymerize and to mix with iron to form more reduced species than might otherwise be expected in this oxygen‐rich system. Indeed, the presence of Fe may aid in the formation of C‐C bonds, as suggested by Belonoshko et al. (2015). Carbon appears to become more siderophile with the increasing pressure, a phenomenon that has been previously reported (Dasgupta et al., 2013; Solomatova et al., 2019). Additionally, the low abundance of C‐Si bonds indicates that carbon is less likely to mix with the silicate than might otherwise be expected. This is suggestive of silicate and carbonate melt immiscibility, which has been well documented in carbonatites (Bodeving et al., 2017; Lee & Wyllie, 1998).

Our work examines a melt with subequal amounts of carbonate, silicate, and metal, differing from previous *ab initio* carbon‐bearing melt studies (Du et al., 2017; Ghosh et al., 2017; Karki et al., 2020; Solomatova et al., 2019). In Figure 4c, we compare the carbon speciation results of this work at 4,000 K directly to the work of Solomatova et al. (2019), which examined a carbon‐bearing pyrolite melt (pyrolite + 3.35 wt% CO), and the work of Karki et al. (2020), which examined a carbon, hydrogen, and iron‐bearing silicate melt, where the volatile species are added in both oxidized and reduced forms. We will thus refer to our system as carbonatitic, the system of Solomatova et al. (2019) as pyrolitic, and the oxidized and reduced systems of Karki et al. (2020) as oxidized silicate and reduced silicate, respectively. The redox conditions of the system play a large role in speciation (Ghosh et al., 2017). The relative redox conditions between four systems mentioned above can be approximated by comparing the C/O ratios. The oxidized silicate melt is the most oxidized composition with a C/O ratio of 0.067 compared to 0.082, 0.083, and 0.170 for the pyrolitic, reduced silicate, and carbonatitic melts, respectively. We examine carbon bonds to the same four elements (carbon, oxygen, iron, and silicon) in all systems. In the case of the oxidized and reduced silicate systems, we have removed the bonds and subsequent coordination to hydrogen atoms for comparison purposes.

Average carbon coordination is similar between the carbonatitic, pyrolitic, and oxidized silicate systems. The reduced silicate system has a larger carbon coordination than the other systems. This is unusual, as average carbon coordination numbers in the reduced silicate system approach a value of 4.5 at high pressures; typically, carbon species at lower mantle pressures adopt a fourfold coordination. The coordination of carbon to each individual element varies widely across systems. In the carbonatitic system, the average coordination to oxygen is lower than in the pyrolitic system, a difference that increases with the depth. Carbon bonding in the oxidized silicate melt consists mostly of carbon‐oxygen bonds. Subsequently, carbon bonding to all other elements is suppressed. Alternatively, in the reduced silicate melt, carbon bonding to oxygen is suppressed, and bonding to all other elements is elevated. All systems show increased carbon‐oxygen coordination numbers with the increasing pressure. Carbon‐silicon coordination is low in all systems but the reduced silicate system, which approaches a coordination number of one at high pressures. Most striking are the differences in carbon‐carbon and carbon‐iron coordination numbers between systems. Due to the greater number of carbon atoms in the carbonatitic system, we would expect to see more carbon‐carbon bonding than in any of the other systems, and this is generally true. However, the carbon‐carbon coordination in the reduced silicate system is very similar despite only 4% of the atoms being carbon versus 9% in the carbonatitic system, indicating that the redox conditions of the system greatly influence carbon’s behavior. Additionally, the average coordination of carbon to carbon increases in all systems but the pyrolitic system, which decreases with pressure. This can be partly explained by the high affinity of carbon for iron in the pyrolitic system; the increase in C‐Fe bonding may inhibit an increase in C‐C bonding. In both the carbonatitic and pyrolitic systems, C‐Fe coordination increases with the pressure. However, the rate of increase is larger in the pyrolitic system. In fact, at 148 GPa, carbon forms on average one extra bond to iron in the pyrolitic system versus the carbonatitic system (1.9 vs. 0.9). The differences in C‐C and C‐Fe bond abundances between the carbonatitic and pyrolitic systems suggest that carbon becomes less siderophilic when in the presence of more carbon and chooses instead to polymerize. This conclusion disagrees with a recent paper by Grewal et al. (2021) that measured carbon partitioning coefficients and found that carbon becomes more siderophile with increasing bulk carbon content of the system. However, these measurements were taken at 3 GPa, and our calculations span a broader range of temperatures. At 3 GPa, C‐Fe bonds are actually more abundant than in the pyrolitic system, and C‐Fe bond abundances in the pyrolitic system only overtake the carbonatitic system around 20 GPa. In the oxidized and reduced silicate systems, carbon‐iron coordination numbers are more scattered but generally invariant with pressure. The coordination number fluctuates around a value of 2.1 in the reduced silicate system and a value of 0.1 in the oxidized silicate system. In general, the carbonatitic system displays a propensity for C‐C bonding that is unmatched by the other systems, likely due to the high number of carbon atoms. The increase in C‐C bonding leads to compensatory drops in C‐O, C‐Fe, and C‐Si bonding as compared to the pyrolitic and oxidized silicate systems. Extrapolating this trend, we expect to see increased C‐C bonding in systems containing more carbon, at the expense of other elements.

**Figure 5:**
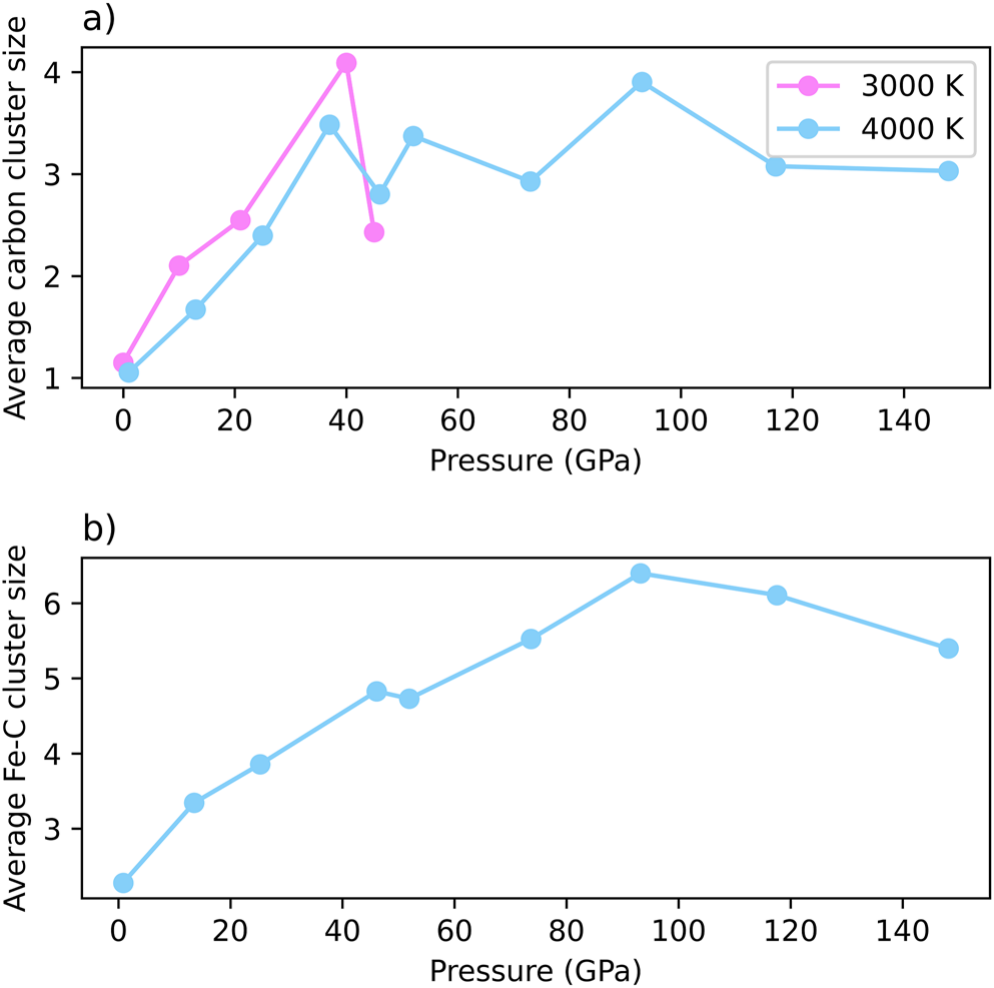
Average cluster sizes as function of pressure and temperature for (a) carbon only clusters and (b) carbon‐iron clusters. Cluster sizes generally increase with increasing pressure.

Due to the high proportion of C‐C bonding in the carbonatitic system, we expanded our speciation analysis beyond the first coordination sphere of individual atoms. Instead, we searched for clusters of carbon atoms, where a cluster consists only of carbon atoms bound together by C‐C bonds. Figure 5a examines the average carbon cluster size as a function of pressure and temperature. In the 4,000 K results, average carbon cluster size rapidly increases from 1.1 carbon atoms at 1 GPa to 3.5 carbon atoms at 37 GPa, in line with the general increase in carbon‐carbon bonding from 1 to 37 GPa (Figure 4a). Beyond 37 GPa, the average carbon cluster size oscillates around a value of 3.2. The 3,000 K results are similar. There is a rapid increase in carbon cluster size from 1.1 carbon atoms at ambient pressure to 4.1 carbon atoms at 40 GPa, suggestive of carbon’s ability to polymerize. Similarly, we examine the size of C‐Fe clusters with pressure in Figure 5b. An iron‐carbon cluster consists only of C‐C and C‐Fe bonds, and must have at least one C‐Fe bond to be considered. Generally, as both C‐Fe and C‐C bonding increase with pressure (Figure 4a), average iron‐carbon cluster size also increases with pressure. The average cluster size increases from an average value of 2.3 at 1 GPa and 4,000 K to an average value of 6.4 at 93 GPa. From 93 to 148 GPa, the average cluster size drops slightly to 5.4 atoms.

**Figure 6:**
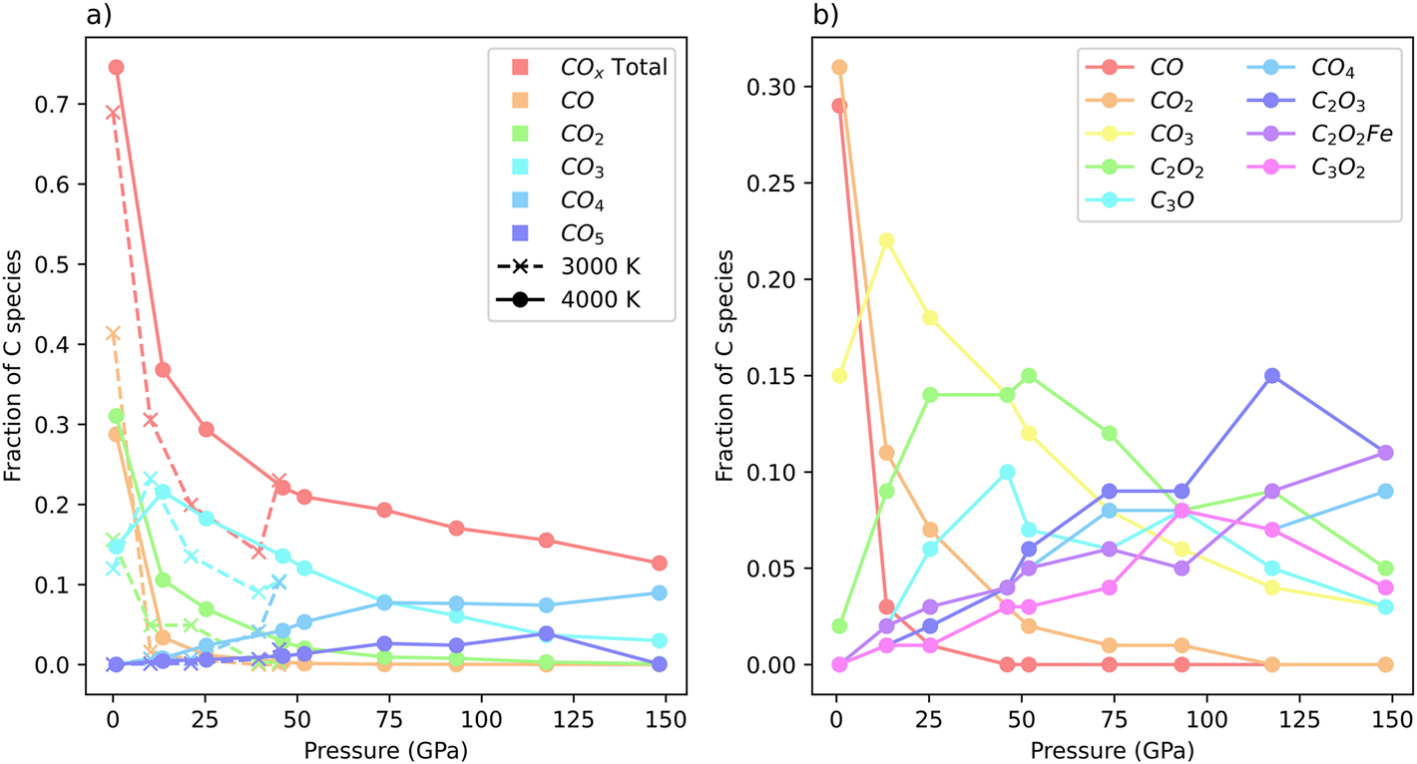
(a) Abundances of carbonate species as a function of pressure and temperature. Individual carbonate coordination states are plotted in addition to the sum total of all carbonate groups (red line). (b) Abundances of the nine most abundant carbon species across all 4,000 K simulations. Higher coordination species are more dominant at higher pressures, regardless of the elemental identity of the bonding atoms.

As C‐C and C‐Fe bond abundances rise with pressure, there is a decrease in carbonate abundance. For the purposes of this analysis, we define a carbonate as a carbon atom bound only to oxygen and to no other element. With this definition, species traditionally not identified as carbonates, such as CO or CO_2_, are included as carbonate species. Figure 6a examines the abundances of carbonates species as a function of pressure and temperature. At the lowest pressure, the majority of carbon species are carbonates (69% at 3,000 K and 75% at 4,000 K). There is a rapid decrease in carbonate fraction with increasing pressure. At 13 GPa and 4,000 K, carbonate species are only 37% of total carbon species. From 13 to 148 GPa, carbonate percentage decreases more slowly to a value of 13%, as carbon increasingly forms bonds to other atoms (Figure 4a). The loss of carbonate species with the increasing pressure indicates that carbon is becoming more reduced with depth. Although the total abundance of carbonates decreases with pressure, the abundances of individual carbonate coordination environments vary. More highly coordinated carbonate groups (such as CO_4_ and CO_5_) are more abundant at higher pressures, while carbonate groups with lower coordination are more abundant at lower pressures. In fact, the relationship between more highly coordinated species and pressure holds for all carbon species, regardless of the coordinating atom. Figure 6b plots the abundances of the nine most common species across all 4,000 K simulations as a function of pressure. Species with higher coordination numbers are more dominant at higher pressures, regardless of the elemental identity of the coordinating atoms. For instance, at 13 GPa the three most dominant species are CO_3,_ CO_2_, and C_2_O_2_, which exhibit either twofold or threefold coordination. At 148 GPa, the three most dominant species are C_2_O_2_Fe, C_2_O_3_, and CO_4_, which all exhibit fourfold coordination.

Similar to our carbon speciation analysis, we evaluate iron (Figure S3, Supporting Information S1) and silicon speciation (Figure S4, Supporting Information S1). Iron and silicon both bond to two elements—oxygen and carbon. For both cations, speciation results are similar at 3,000 K and 4,000 K. From 1 to 148 GPa at 4,000 K, the abundance of C‐Fe bonds increases from 4% to 10%, while the abundance of Fe‐O bonds decreases from 96% to 90% (Figure S3a, Supporting Information S1). The percentage of Si‐C bonds increases from 0.8% at 1 GPa to 4% at 37 GPa, then decreases to 2% at 148 GPa and 4,000 K, with the balance taken up by Si‐O bonds (Figure S4a, Supporting Information S1). The speciation results are also presented in coordination space (Figures S3b and S4b, Supporting Information S1) and compared to the results from statistical sampling (Figures S3a and S4a, Supporting Information S1). Fe‐O bonding is more prevalent, and C‐Fe bonding is less prevalent than expected from statistical sampling. The discrepancy between expected and actual results decreases with the increasing pressure, as C‐Fe abundance increases. Si‐O bonding is also more prevalent while Si‐C bonding is less prevalent than expected. This discrepancy increases with the increasing pressure.

### 3.3 Average Coordination Numbers

In a silicate melt, cation‐oxygen bonds are typically the only bonds considered when evaluating coordination states (Ghosh et al., 2017; Solomatova & Caracas, 2019), as oxygen is the most prominent bonding anion in the system. However, due to the complicated bonding in our carbon‐rich system, we evaluate cation coordination to all bonding atoms, not just to oxygen.

**Figure 7:**
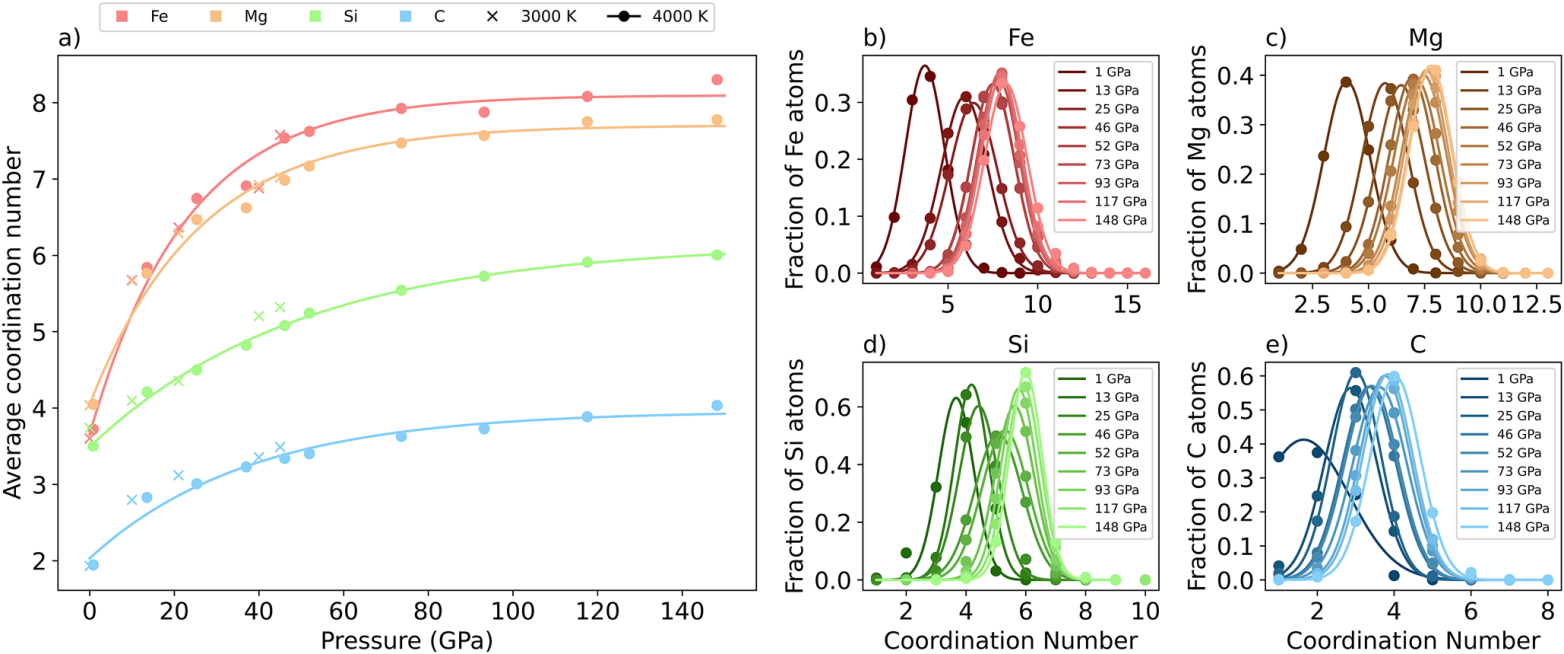
(a) Average cation coordination number as a function of pressure and temperature. (b)**–**(e) Coordination state distributions for (b) iron, (c) magnesium, (d) silicon, and (e) carbon at 4,000 K from 1 to 148 GPa. All distributions are fit to a Gaussian profile and shift to higher coordination numbers with increasing pressure.

The average coordination state of all cations increases with pressure (Figure 7a). Iron’s average coordination number increases from 3.7 to 8.3 from 1 to 148 GPa at 4,000 K. Over the same conditions, magnesium’s coordination number increases from 4.0 to 7.8. For both elements, coordination increases rapidly from 0 to 45 GPa, then levels off. Silicon’s coordination increases gradually from tetrahedral at low pressure to octahedral at high pressure as expected from previous experimental (Williams & Jeanloz, 1988; Xue et al., 1989) and computational (de Koker et al., 2008; Ghosh et al., 2014; Karki et al., 2007) work on silicate melts and glasses. Carbon’s coordination increases most rapidly from 1 to 13 GPa, where it evolves from twofold coordination to triangular coordination as it transitions from a molecular species to part of the liquid framework. Beyond 13 GPa, coordination increases gradually from triangular coordination to tetrahedral coordination, mirroring the sp^2^‐sp^3^ transition found in solid carbonates in the lower mantle (Boulard et al., 2015; Oganov et al., 2008). The 3,000 K results closely follow the 4,000 K results, and exhibit similar trends with pressure.

Figures 7(b)–7(e) plots the distribution of coordination states for iron, magnesium, silicon, and carbon at 4,000 K. Generally, the distributions fit a Gaussian profile and shift to higher coordination numbers with increasing pressure. The broadness of the distribution, measured by the full width at half maximum (FWHM) value, is an indication of the number of simultaneous coordination states and a proxy for the cation’s preference for the central coordination number. Iron and magnesium have similar coordination distributions. Iron has the highest coordination numbers and the broadest distributions overall. For example, the FWHM value at 4,000 K and 117 GPa for iron is 2.70 versus 2.28, 1.53, and 1.36 for magnesium, carbon, and silicon respectively. Silicon coordination distributions are narrow when centralized around coordination numbers of 4 and 6 (FWHM values of 1.35 and 1.28), indicating its preference for regular coordination polyhedra. The distributions broaden when centralized around a coordination number of 5 (FWHM of 1.89). Carbon is the smallest cation, and therefore has the lowest coordination numbers. The coordination distributions are narrowest when centralized around threefold and fourfold coordination (FWHM values of 1.51 and 1.54), and broadest when transitioning between twofold and threefold coordination and between threefold and fourfold coordination (FWHM of 2.78 and 1.65). Coordination distributions are affected by temperature and pressure (Figure S5, Supporting Information S1). For all cations, distributions broaden with increasing temperature as greater thermal energy allows more simultaneous coordination states to be accessed at a given pressure. On average, FWHM values for all cations increase by 0.27 when the temperature is increased from 3,000 to 4,000 K. For iron, the average coordination number slightly increases with temperature. Magnesium, silicon, and carbon’s average coordination numbers slightly decrease with increasing temperature. A similar effect is observed in pyrolite melts (Solomatova & Caracas, 2019).

**Figure 8:**
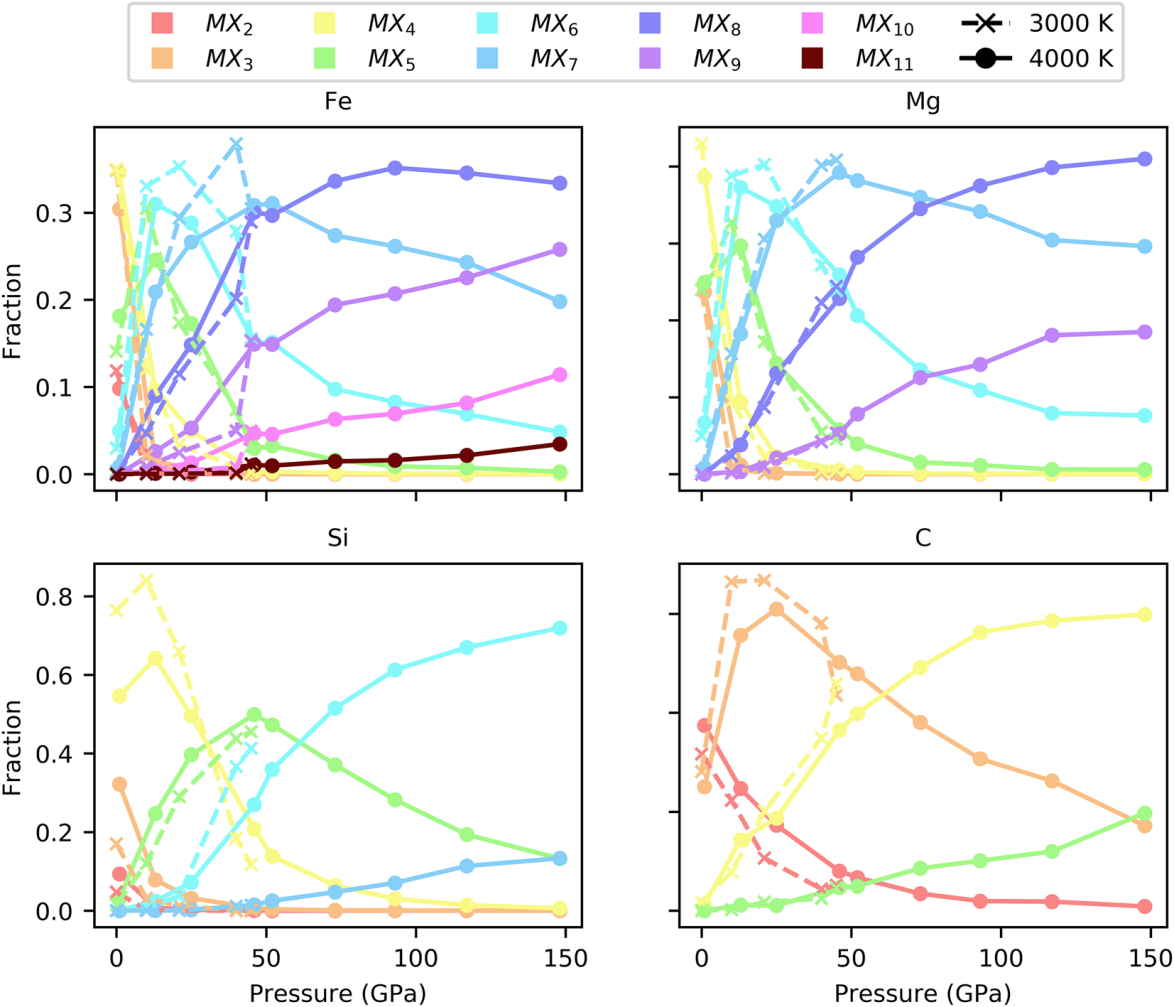
Abundances of coordination states as a function of pressure and temperature. Coordination states are color coded by the legend, where M indicates the cation of interest and X indicates a coordinating anion. For clarity, only two to eleven‐fold coordination states are plotted. More highly coordinated cations are more abundant with increasing pressure. Most of the coordination changes occur in the first 50 GPa.

Coordination changes in the simulated melt are continuous. Silicon coordination at 4,000 K, for example, continuously increases from dominantly fourfold from 0 to ∼30 GPa, to fivefold from 30 to ∼60 GPa, and finally to sixfold from 60 GPa to >148 GPa (Figure 8). Similarly, carbon coordination increases continuously from predominantly twofold from 0 to ∼1 GPa, to threefold from 1 to 60∼ GPa, to fourfold from 60 to >148 GPa. Iron and magnesium both jump from predominantly fourfold coordination at 1 GPa to predominantly sixfold coordination at 13 GPa, but their coordination increases are continuous from sixfold to eightfold coordination. The continuous coordination changes in the melt are distinct from many glasses and solids, which tend to skip coordination states to achieve a preferred coordination at a given pressure and temperature. For instance, in many silicates in the mantle, silicon adopts a combination of fourfold and sixfold coordination states, rather than adopt a fivefold coordination state (Finger & Hazen, 2000; Xue et al., 1989). For instance, carbon coordination at 52 GPa is 7% twofold, 48% threefold, 40% fourfold, and 5% fivefold.

**Figure 9:**
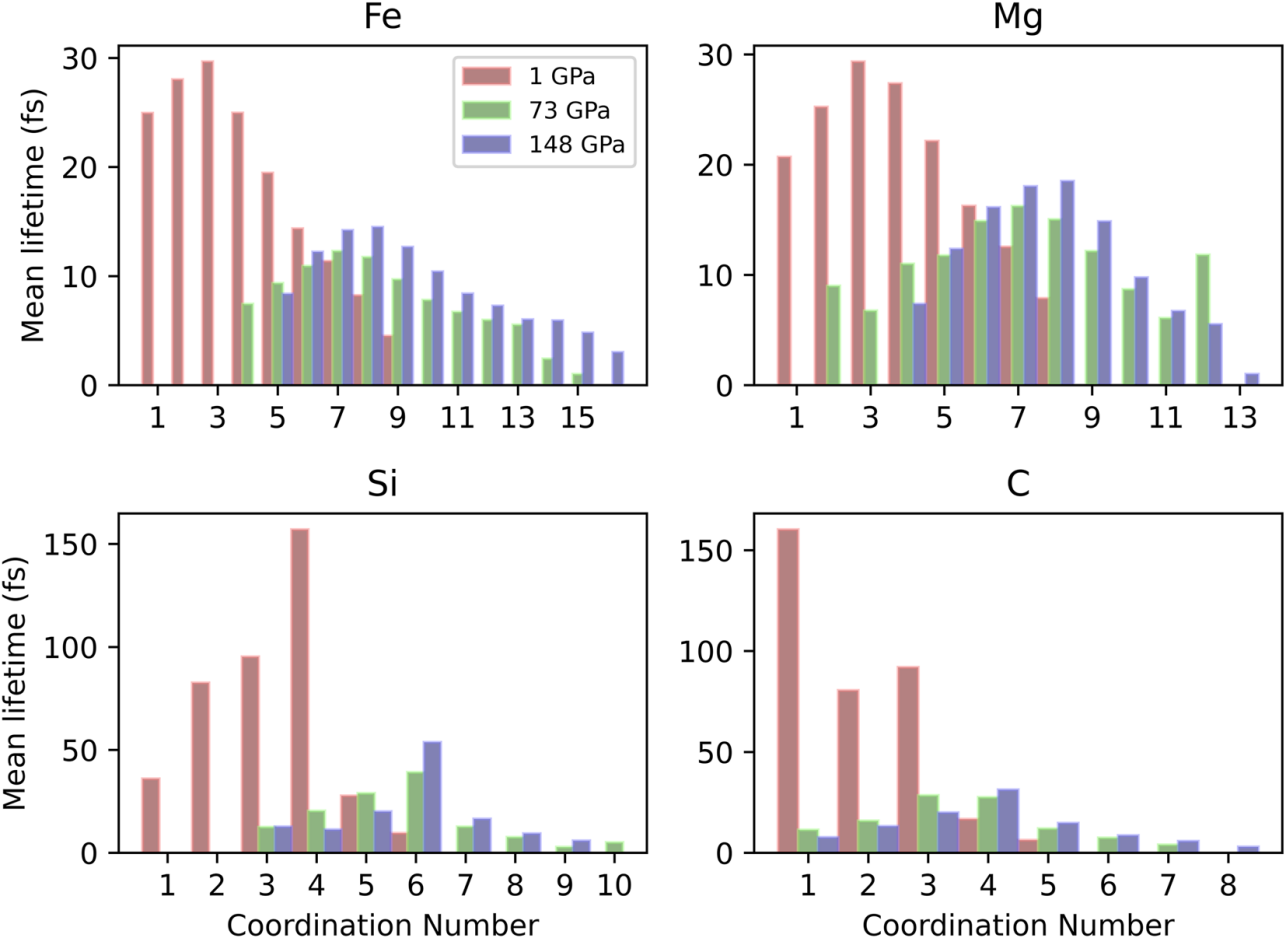
Average coordination state lifetimes in femtoseconds for (a) iron, (b) magnesium, (c) silicon, and (d) carbon at 1 GPa (red), 73 GPa (green), and 148 GPa (purple). With increasing pressure, lifetime distributions shift to higher coordination numbers and mean lifetimes decrease.

Mean coordination lifetimes at 4,000 K are plotted in Figure 9. The lifetime of a specific coordination state is an indication of both its overall stability and the role of the cation as either a network former or a network modifier. Longer lifetimes typically indicate a network former, as the cation is largely unperturbed in the melt network, while shorter lifetimes indicate a network modifier. Coordination lifetime distributions are heavily skewed right (Figures S6–S8, Supporting Information S1) but tend to have the same shape, suggesting that the mean coordination lifetime is a valid comparison of lifetime stability between cations. With the increasing pressure, lifetimes shift to increasingly higher coordination states, indicating that at higher pressures, higher coordination numbers are more stable. However, the lifetimes of the coordination environments generally decrease with the increasing pressure. Most of the changes in the lifetime distributions occur between 1 and 73 GPa, where the majority of coordination changes occur. Between 73 and 148 GPa, there are few changes in the lifetime distribution, matching the general flattening in the average coordination number for all cations at these pressures (Figure 7a). Silicon and carbon species have the longest lifetimes overall, but at 73 and 148 GPa, their average lifetimes are similar to those of iron and magnesium. Their relatively long lifetimes at low pressure indicate that they tend to be network formers at these conditions, as seen in previous studies on silicate melts and glasses (Kubicki & Stolper, 1995; Lesher et al., 1996). Note that CO and CO_2_ are molecular species that do not integrate into the melt network, but represent most of the CX_1_ and CX_2_ species in Figure 9. Thus, these lifetimes indicate their stability as molecular species rather than their role as network formers. However, CX_3_ species do integrate into the melt network and have long average lifetimes compared to iron and magnesium species (92 fs), indicating that carbon plays a role as a network former. The longest lifetimes of silicon‐bearing coordination environments are 157, 39, and 54 fs at 1, 73, and 148 GPa respectively. The longest lifetimes of carbon‐bearing coordination environments are 160, 28, and 31 fs at the same conditions. Iron‐bearing and magnesium‐bearing coordination environments generally have shorter lifetimes, indicating that they behave more like network modifiers. The longest lifetimes of iron‐bearing coordination environments are 30, 12, and 14 fs, and the longest lifetimes of magnesium‐bearing coordination environments are 29, 16, and 18 fs at 1, 73, and 148 GPa, respectively. Iron and magnesium have similar lifetimes across all pressures shown in Figure 9, whereas silicon and carbon have much longer lifetimes at 1 GPa compared to 73 and 148 GPa.

Overall, the simulations reveal that with the increasing pressure, the melt evolves to a denser, more highly coordinated structure whose cations are rapidly exchanging nearest neighbors. The coordination polyhedra become increasingly transient species, while maintaining a relatively narrow spread of coordination numbers.

### 3.4 Redox Conditions

**Figure 10:**
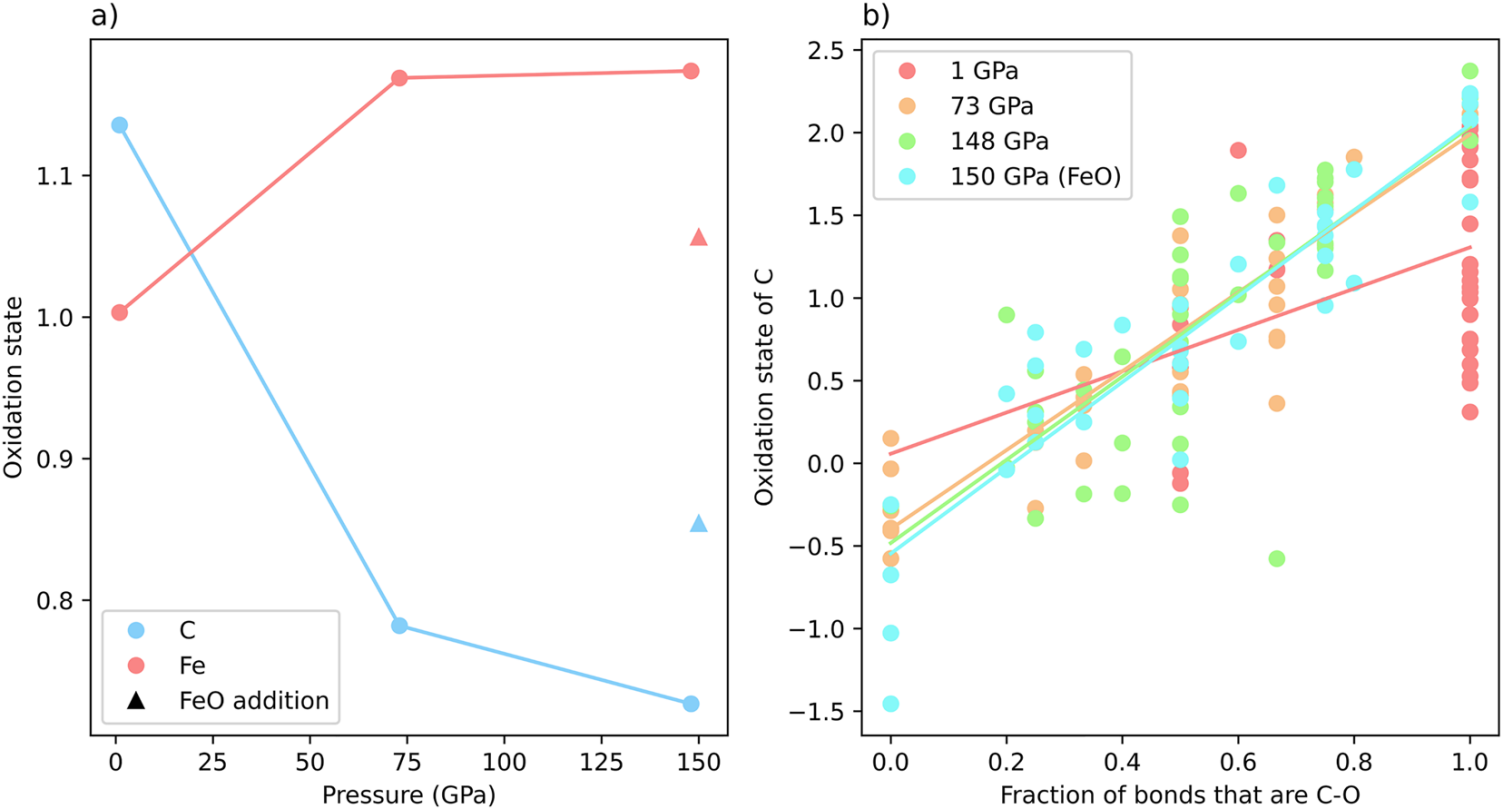
(a) Average oxidation states of carbon and iron as a function of pressure. With increasing pressure, carbon becomes more reduced and iron becomes more oxidized. The addition of four FeO units (triangles) reduces iron and oxidizes carbon relative to the non‐oxidized simulation. (b) Oxidation sates of individual carbon atoms as a function of fraction of bonds that are C‐O bonds. Oxidation states of carbon increase linearly with increasing fraction of C‐O bonds.

To see how differing redox conditions affect melt behavior, we ran an additional simulation with an oxidized starting composition at 150 GPa and 4,000 K (see Section 2). FeO was added to the starting composition by replacing four Mg atoms with four Fe atoms to start with a mix of iron metal and FeO. The results are presented in Figure S9, Supporting Information S1. Generally, speciation and coordination results are the same in both systems, with a few differences. C‐Fe bonding is more abundant and C‐C bonding is less abundant in the oxidized system (Figure S9b, Supporting Information S1), although this observation can be explained by the increased number of Fe atoms in the system. Total carbonate percentage is slightly increased in the oxidized system (15% vs. 13%), due to an increase of CO_4_ groups. Additionally, we examined the effect of both pressure and redox conditions on the oxidation states of the elements in the simulation. Bader volumes were calculated, and Bader charges were integrated using the Bader charge analysis algorithm (Henkelman et al., 2006; Sanville et al., 2007; Tang et al., 2009; Yu & Trinkle, 2011). Oxidation states of all five elements were found. Oxygen, silicon, and magnesium are essentially invariant with pressure, with average oxidation states of −1.4, 2.9, and 1.6, respectively. Due to redox exchange between iron and carbon, iron and carbon have oxidation states that vary with pressure (Figure 10a). With the increasing pressure, carbon is reduced by iron. Iron’s average oxidation state rises from 1.0 at 1 GPa to 1.2 at 73 GPa while carbon’s average oxidation state falls from 1.1 at 1 GPa to 0.8 at 73 GPa. This change in oxidation state is evidenced not only by carbon’s predilection to form reduced C‐C and C‐Fe species but also by the overall decrease in carbonate groups (Figure 6a). Carbonate stability is particularly sensitive to the redox conditions of the system, and carbonates are typically only stable in oxidizing environments (Stagno et al., 2011, 2015). Finally, we examined how the addition of four FeO units affects the oxidation states of iron and carbon. The oxidation state of carbon increases with increasing oxygen fugacity. Carbon’s oxidation state rises from 0.7 in the reduced composition to 0.9 in the oxidized composition, and iron’s oxidation state falls from 1.2 in the reduced composition to 1.1 in the oxidized composition. Thus, the oxidation states of carbon and iron are dependent on the redox conditions of the system, and this dependency is manifested in the speciation results (Figure S9b, Supporting Information S1). Figure 10a plots the oxidation states of carbon and iron averaged across all carbon and iron atoms. However, each individual atom has an oxidation state that is dependent on its bonding environment. Figure 10b examines the oxidation states of individual carbon atoms as a function of the fraction of bonds it forms that are C‐O bonds. Generally, oxidation states are higher for carbon atoms with more carbon‐oxygen bonds. This trend is linear, and the trend lines at 73, 148 GPa, and 150 GPa (FeO simulation) are in good agreement. At 1 GPa, there is more scatter in the data, due to the abundance of molecular CO and CO_2_ groups. With the increasing pressure, the abundance of carbon‐oxygen bonds decreases, and the abundance of carbon bonds to other elements increases (Figure 4a). Therefore, within melts of similar composition at increasing depth in the Earth, carbon oxidation states generally shift to the left side of the plot in Figure 10b, down the trendline, to yield lower average carbon oxidation states and more reduced carbon overall.

### 3.5 Diffusivities

**Figure 11:**
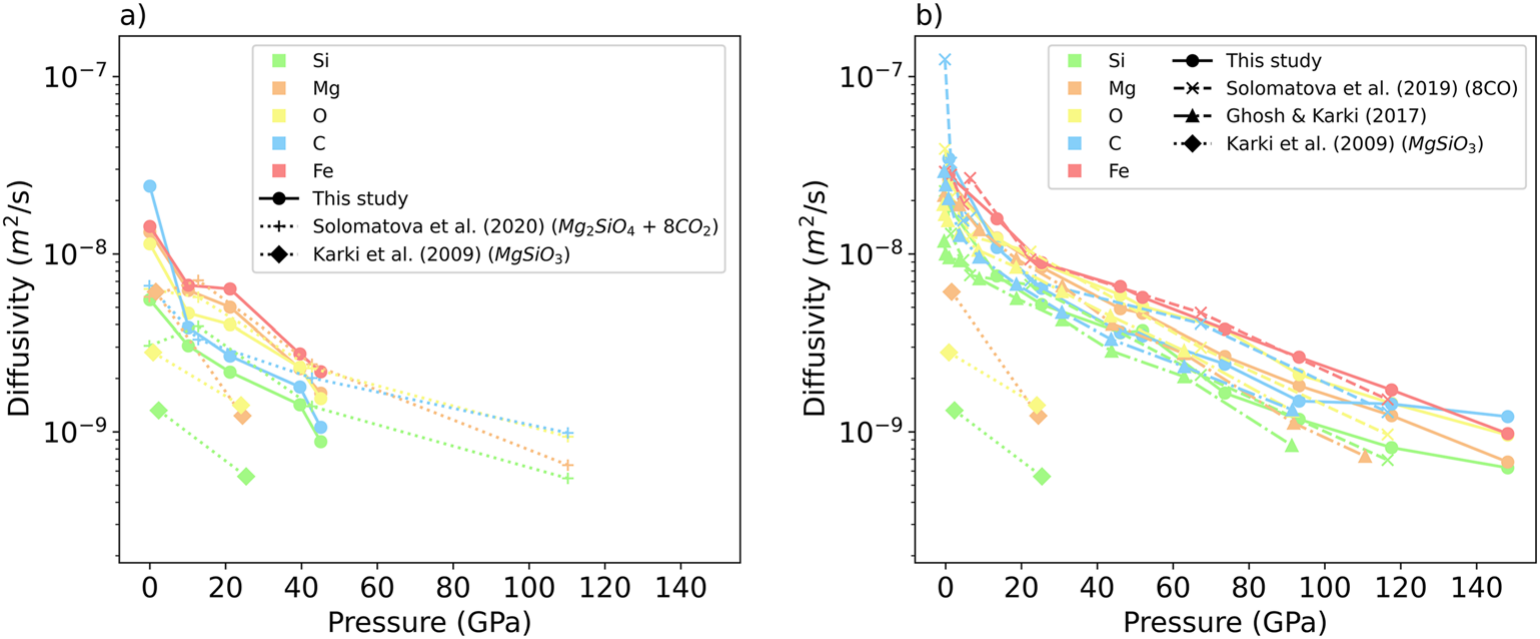
Diffusivities of silicon, magnesium, oxygen, carbon, and iron at (a) 3,000 (k) and (b) 4,000 K as a function of pressure. Values from this study (solid lines, circles) are compared to Solomatova et al. (2020) (dotted lines, plusses), Solomatova et al. (2019) (dashed lines, x’s), and Ghosh and Karki (2017) (dashed dotted line, triangles). Diffusivities increase with temperature and decrease with pressure.

The diffusion coefficients of all elements are plotted at 3,000 K in Figure 11a and at 4,000 K in Figure 11b. Diffusion coefficients increase with increasing temperature and decrease with increasing pressure, as expected. The diffusivity of an individual element is an indication of its role in the melt. Less mobile elements tend to be network formers, while more mobile elements tend to be network modifiers. Generally, silicon is the least mobile element, followed by carbon, magnesium, oxygen, and iron. Silicon and carbon are network formers, while iron is a network modifier. Carbon's relative diffusivity compared to the other elements changes with pressure. At 1 GPa, carbon is the most mobile element, as it is mostly in molecular species. From 13 to 97 GPa, carbon is the second least mobile element, and from 97 to 148, carbon's diffusivity rises relative to the other elements, indicating its changing role in the melt network. Comparing this study to other carbon‐bearing silicate compositions, we find similar results. At 3,000 K, this study is in good agreement with CO_2_‐bearing olivine melt (Solomatova et al., 2020). At 4,000 K, we find that carbon is less mobile in this study than in CO‐bearing pyrolite (Solomatova et al., 2019), and similar to CO_2_‐bearing MgSiO_3_ (Ghosh & Karki, 2017). The difference between this work and Solomatova et al. (2019) can be explained by the degree of carbon polymerization in the melt. In this work, carbon forms large clusters, which are less mobile. Based on this reasoning, we would expect a melt of this composition to be more viscous than a CO‐bearing pyrolite melt. However, when comparing carbon‐bearing melts to a pure MgSiO_3_ melt (Karki et al., 2009), all cations are more mobile, indicating that carbon‐bearing melts are less viscous than their carbon‐free counterparts. As incorporation of volatile species into silicate melts has been shown to lower viscosity (Ghosh & Karki, 2017), this result is expected.

The diffusivity of an element can be used as a proxy for the homogeneity of the melt. Melts with high diffusivities are more likely to be well mixed. By transforming the carbon diffusion coefficients at 4,000 K into length scales, we can estimate the distance carbon atoms will travel in 1 million years. At 0 GPa, carbon atoms travel 1042, 1912, and 879 m in the carbonatitic, pyrolitic, and CO_2_‐bearing MgSiO_3_ melts, respectively. These numbers indicate that carbonatite melts are less homogenous than carbon‐bearing pyrolite melts, and similarly homogenous compared to CO_2_‐bearing MgSiO_3_. At 136 GPa, the estimated distances drop significantly, with carbon atoms traveling 202, 202, and 98 m in the carbonatitic, pyrolitic, and CO_2_‐bearing MgSiO_3_ melts, respectively. At core‐mantle boundary pressures, the carbonatitic and pyrolitic melts are expected to be similarly homogenous, while the CO_2_‐bearing MgSiO_3_ melt is expected to be less so. We would anticipate that all melts are less well‐mixed at higher pressures, due to the decrease in diffusivity with pressure.

### 3.6 Electronic Structure of the Melt

**Figure 12:**
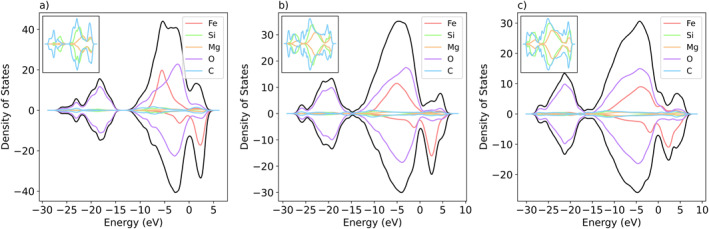
Densities of states at (a) 1 GPa, (b) 73 GPa, and (c) 148 GPa and 4,000 K The total DOS is plotted in black, and individual element components are plotted according to the legend. The inset (top left) zooms in on the silicon, magnesium, and carbon components for clarity. Positive DOS values are contributions from spin up electrons, and negative values are contributions from spin down electrons. At all studied pressures, electrons have energy above the Fermi level (0 eV in the plot), indicating a conducting liquid.

With a significant iron component in our melt (∼10 atomic % Fe), we examine the electronic density of states (DOS) to investigate conductivity behavior in the melt. Figure 12 plots the density of states at 1, 73, and 148 GPa and 4,000 K. The melt is conducting at all pressures examined, even 1 GPa, due primarily to the high iron content. With increasing pressure, the occupancy of the Fermi level increases.

The spin decomposition of the electronic density of states for all the elements, except for iron, shows a clear complete pairing of electrons. There is equal contribution from the spin up and the spin down electrons. Iron, however, is asymmetric at all pressures examined. The uneven occupation of energy levels between spin up and spin down electrons indicates that the iron atoms preserve a local remnant magnetic moment in the melt. The value of the local magnetization is largest at 1 GPa, where the iron curves have the most asymmetry. With the increasing pressure, the asymmetry decreases, and iron becomes less spin polarized, although some magnetization is still apparent even at 148 GPa.

The spin state of iron has important implications for the physical properties of melts and minerals. Spin transitions from high‐spin to low‐spin states in iron‐bearing mantle compositions lead to densification at high pressures (Karki et al., 2018; Liu et al., 2020; McCammon et al., 2013; Speziale et al., 2005). Local spin state affects iron coordination and bond length in melts, with high‐spin iron atoms being more highly coordinated with longer bonds (Ghosh & Karki, 2020). Additionally, the spin state of iron is known to affect the partitioning behavior of iron between solids and silicate melts (Maeda et al., 2017; Prescher et al., 2014). Previous work on iron spin states in melts and glasses disagrees on whether or not a spin transition occurs, and the completeness of that transition. Mossbauer and X‐ray emission studies of silicate glasses find no evidence of a spin transition below 84 GPa (Lee, 2014; Mao et al., 2014; Prescher et al., 2014). However, higher pressure Mossbauer, X‐ray emission, and nuclear forward scattering studies (up to 135 GPa) of silicate glasses indicate that a broad, partial spin transition may occur at higher pressures (Gu et al., 2012; Maeda et al., 2017). Using *ab initio* molecular dynamics, Karki et al. (2018) finds that a spin transition occurs gradually beginning around 100 GPa in silicate melts. In our work, the local magnetization on the iron atoms flips more often between spin up and spin down with the increasing pressure. At 93 GPa, the magnetization begins to flip between positive and negative values, suggesting the onset of a gradual spin transition. Our prediction that a spin transition initiates at about 93 GPa agrees with experimental measurements of a spin transition beginning at roughly 100 GPa (Gu et al., 2012; Maeda et al., 2017). At higher pressures, the flipping exhibits transient non‐magnetized states that last for a few femtoseconds (Figure S10, Supporting Information S1).

## 4 Conclusions

These simulations of a carbonate‐silicate‐metal melt highlight the important effect of high carbon content, which greatly affects the chemical and physical properties of the melt. Melts with high carbon contents have unusual properties, including low viscosities (Stagno et al., 2018), high electrical conductivities (Gaillard et al., 2008), and depressed melting temperatures (Ghosh et al., 2009), which are expressed in carbonatite melts in the upper mantle and could be expressed in carbon‐rich melts in the lower mantle. Carbon‐rich melts could serve as parent melts for intriguing carbon‐bearing species such as diamonds or iron‐carbides. Additionally, this work examines the interaction of a carbon‐bearing silicate melt with metal, which serves as an example of the types of complicated melt compositions we may expect to see in the lower mantle and at the core‐mantle boundary. The structure of the melt is evaluated, with a particular emphasis on coordination and speciation.

Coordination numbers in the simulated melts increase continuously with pressure, leading to unusual coexisting coordination environments. Coordination distributions broaden around non‐preferred coordination states and with increasing temperature. Coordination states are long‐lived at low pressure, and short‐lived at high pressure, indicating the changing nature of the melt network with pressure. With increasing pressure, species become more transient and bonds are broken and formed more quickly. Thus, we would expect carbonate‐silicate‐metal melts to be more reactive with surrounding species with increasing depth in the Earth. Silicon and carbon have the longest‐lived coordination states, indicating their role as network formers. Diffusion coefficients decrease with pressure and increase with temperature. Silicon and carbon generally have the lowest diffusivities, aligning with the previous idea that they are network formers. This volatile‐bearing melt has a lower viscosity than pure silicate melts (Karki et al., 2009), indicating that volatile‐bearing melts are more mobile and could serve to transport or concentrate certain elements that partition into the melt. In particular, rare earth elements have been shown to concentrate in carbonatites (Cullers & Medaris, 1977; Hou et al., 2015; Yang et al., 2003). Bond lengths in the melt generally decrease with pressure, although increases are also observed when associated with coordination changes in the cations. Melts are conducting at all pressures, and iron has some magnetization, which diminishes with pressure. We find evidence for a spin transition in the iron atoms that begins around 90 GPa. The spin transition will change the compressibility of the melt, and allow for increased densification of the melt structure. Speciation is complicated, and many unusual species are present in the carbonate‐silicate‐metal melts. The complicated nature of the melt stems largely from carbon’s ability to behave as both a cation and an anion. Carbon forms bonds to all elements except magnesium, and begins to replace oxygen in the melt network with the increasing pressure. Carbon's replacement of oxygen rather than silicon atoms allows more informed predictions about the types of carbon‐bearing species expected in carbon‐bearing melts in the deep Earth. For instance, we anticipate more reduced carbon species, such as carbon polymers and carbon‐iron clusters, rather than the oxidized carbon species one would expect if carbon replaced silicon instead of oxygen in the melt network. Carbon bonding is highly dependent on both pressure and oxygen fugacity. With the increasing pressure, C‐O bonding becomes less dominant and is compensated by increases in C‐C and C‐Fe bonding. Additionally, C and Fe undergo redox exchange, and carbon oxidizes iron to become more reduced. As a result, carbon‐carbon and carbon‐iron clusters grow, and carbonate abundance decreases. In a more oxidized system, carbon reduces iron, forming more oxidized species overall. Compared to previous work (Karki et al., 2020; Solomatova et al., 2019), our system has greater C‐C bond abundances, indicating a propensity for carbon to bond with itself in carbon‐rich melts at the expense of other types of bonding. Carbonatitic melts may be a parent melt for diamond formation or, coupled with the increasingly siderophile nature of carbon with increased pressure, for a dense C‐Fe liquid.

## Supporting information

Supporting Information S1

## Data Availability

All speciation data presented in this manuscript are publicly available at https://doi.org/10.5281/zenodo.5527283.
