# Supplementary material for: The Speciation and Coordination of a Deep Earth Carbonate‐Silicate‐Metal Melt
Source: J Geophys Res Solid Earth. 2022 Mar 20;127(3):e2021JB023314. doi: 10.1029/2021JB023314 (PMC9286813; doi:10.1029/2021JB023314)
Supplement: Supplementary file 1 — Supporting Information S1 [file JGRB-127-0-s001.docx]

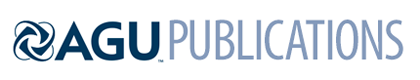


*Journal of Geophysical Research: Solid Earth*

Supporting Information for

**The Speciation and Coordination of a Deep Earth Carbonate-Silicate-Metal Melt**

A. H. Davis^1^, N. V. Solomatova^2^, A. J. Campbell^1^, and R. Caracas^2,3^

^1^Department of the Geophysical Sciences, University of Chicago, 5734 S. Ellis Avenue, Chicago, Illinois 60637, U.S.A., ^2^ CNRS, Ecole Normale Supérieure de Lyon, Laboratoire de Géologie de Lyon LGLTPE UMR5276, Centre Blaise Pascal, Lyon, France., ^3^The Center for Earth Evolution and Dynamics (CEED), University of Oslo, Oslo, Norway.

**Contents of this file**

Figures S1 to S9

**Introduction**

Here we show examples of the mean squared displacements of the atoms at various pressure and temperature conditions (Figure S1). We also show an example of a pair distribution function used for fitting bond distances (Figure S2). We also include speciation results for iron and silicon (Figures S3 and S4). We show how coordination distributions change with temperature (Figure S5) and include coordination species lifetimes at different pressure (Figures S6-S7). Finally, we show how speciation and coordination results change with an oxidized starting composition (Figure S9), and show the magnetic moments of the iron atoms across different pressures (Figure S10).


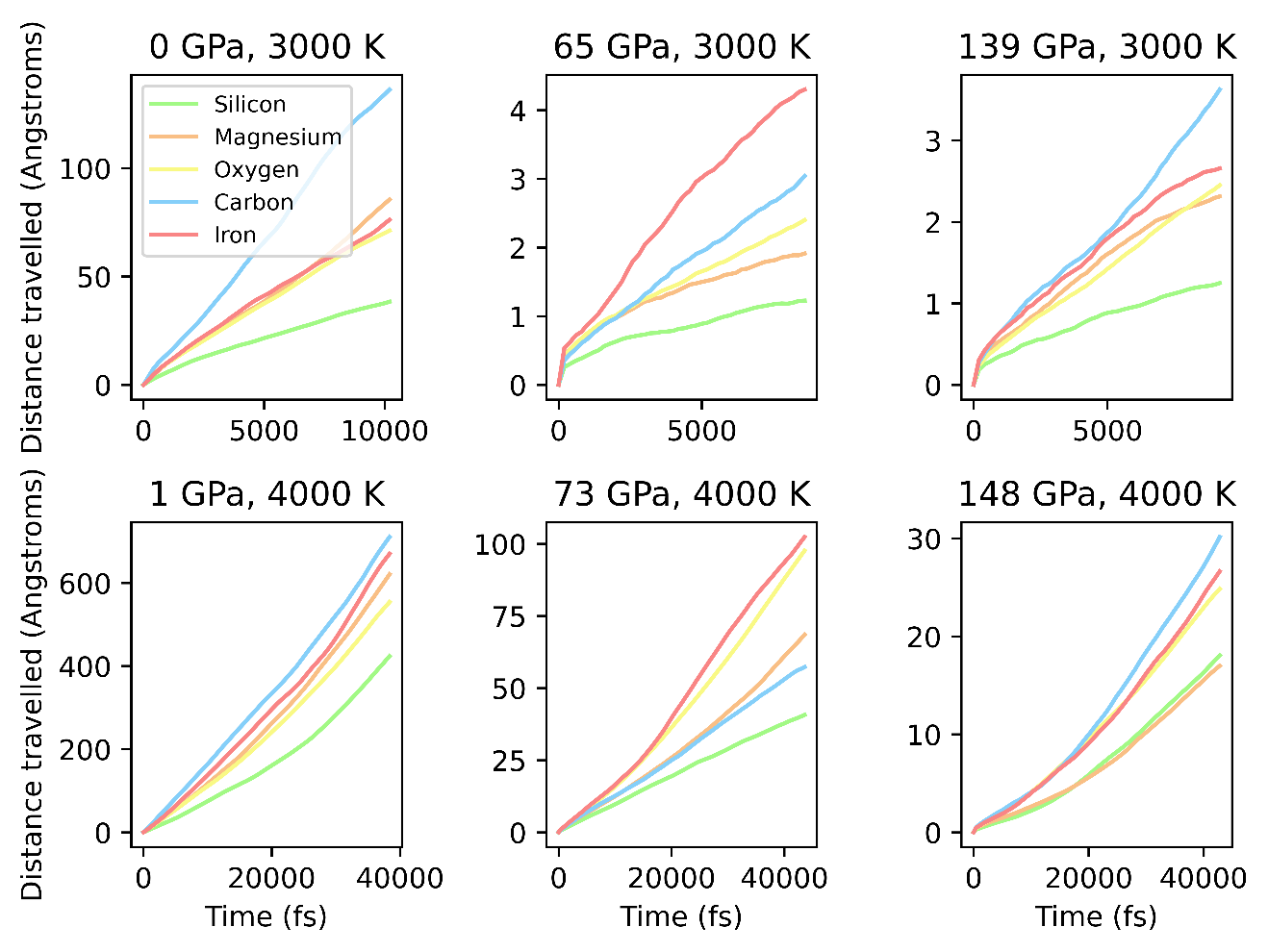


**Figure S1.** Mean squared displacements of the atoms over the course of a simulation at various pressure and temperature conditions. All simulations at 4,000 K show atomic displacements that are steadily increasing, indicating the simulation is molten. 3,000 K simulations above 45 GPa show atomic displacements that level off with increasing time, indicating a glass-like rather than a molten state, in addition to very low mean squared displacement values. These simulations are not included in the final results.


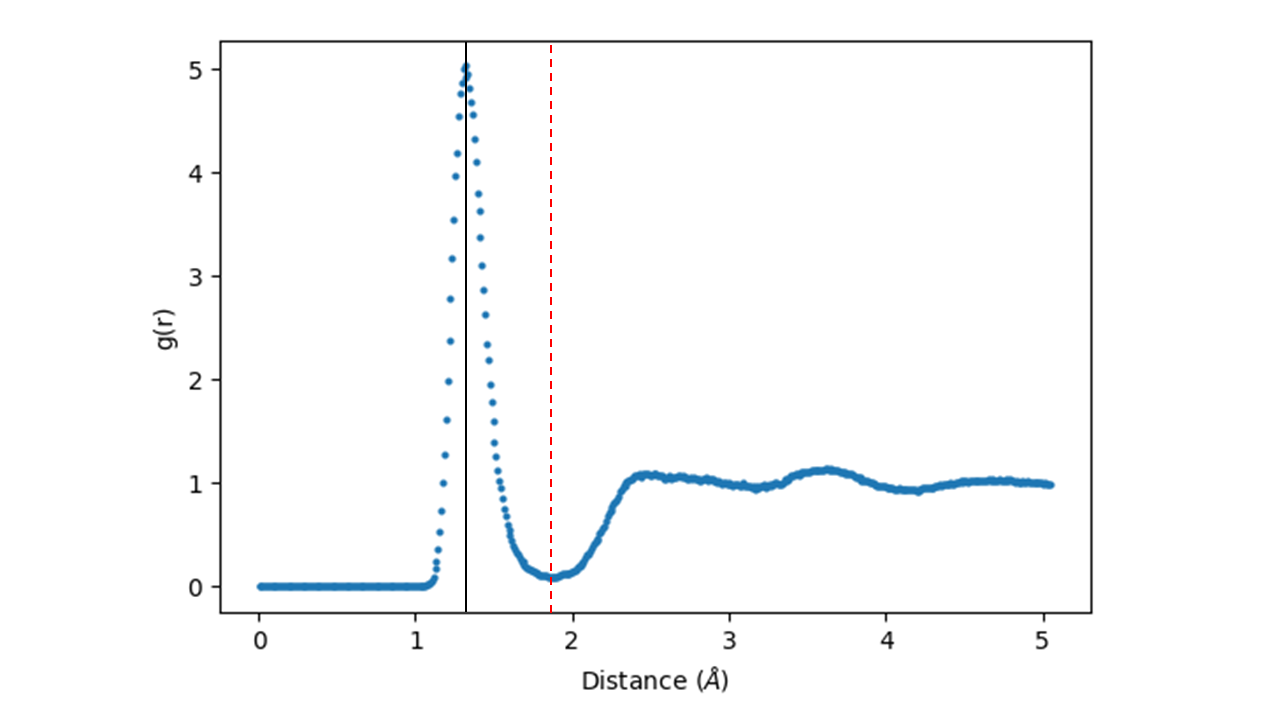


Figure S2. The pair distribution function of C-O at 3,000 K and 45 GPa. The solid black line marks the maximum in the pair distribution function and corresponds to the average bond length for C-O. The dashed red line marks the first minimum in the pair distribution function and corresponds to the maximum acceptable bond length to be considered a C-O bond. First minimums are used in speciation and coordination analysis to define a bond.


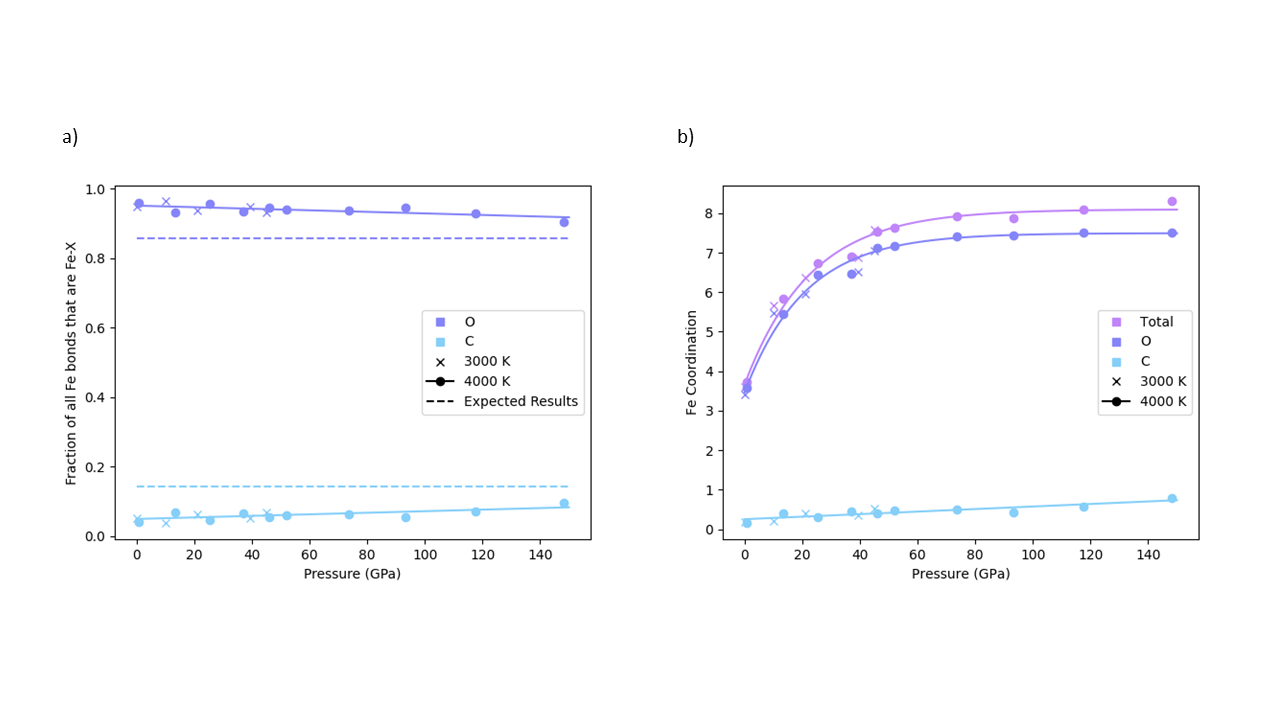


Figure S3. Iron speciation as a function of pressure. a) Displays the fraction of all iron bonds in a simulation that are Fe-O (purple) and C-Fe (blue) bonds. Dashed lines represent the results from statistical sampling. Fe-O bonding is more abundant than expected while C-Fe bonding is less abundant. b) Displays the average coordination number of iron to oxygen (purple), carbon (blue), and to all elements (lilac).


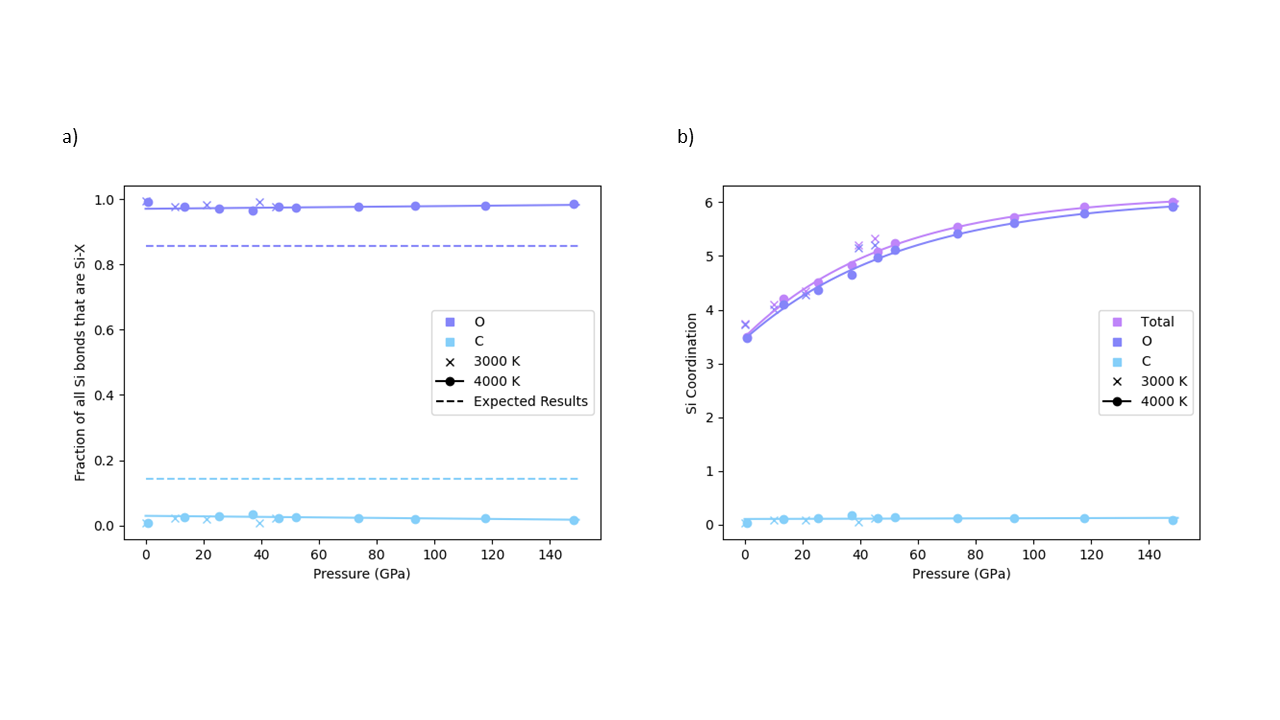


Figure S4. Silicon speciation as a function of pressure. a) Displays the fraction of all silicon bonds in a simulation that are Si-O (purple) and Si-C (blue) bonds. Dashed lines represent the results from statistical sampling. Si-O bonding is more abundant than expected while Si-C bonding is less abundant. b) Displays the average coordination number of silicon to oxygen (purple), carbon (blue), and to all elements (lilac).


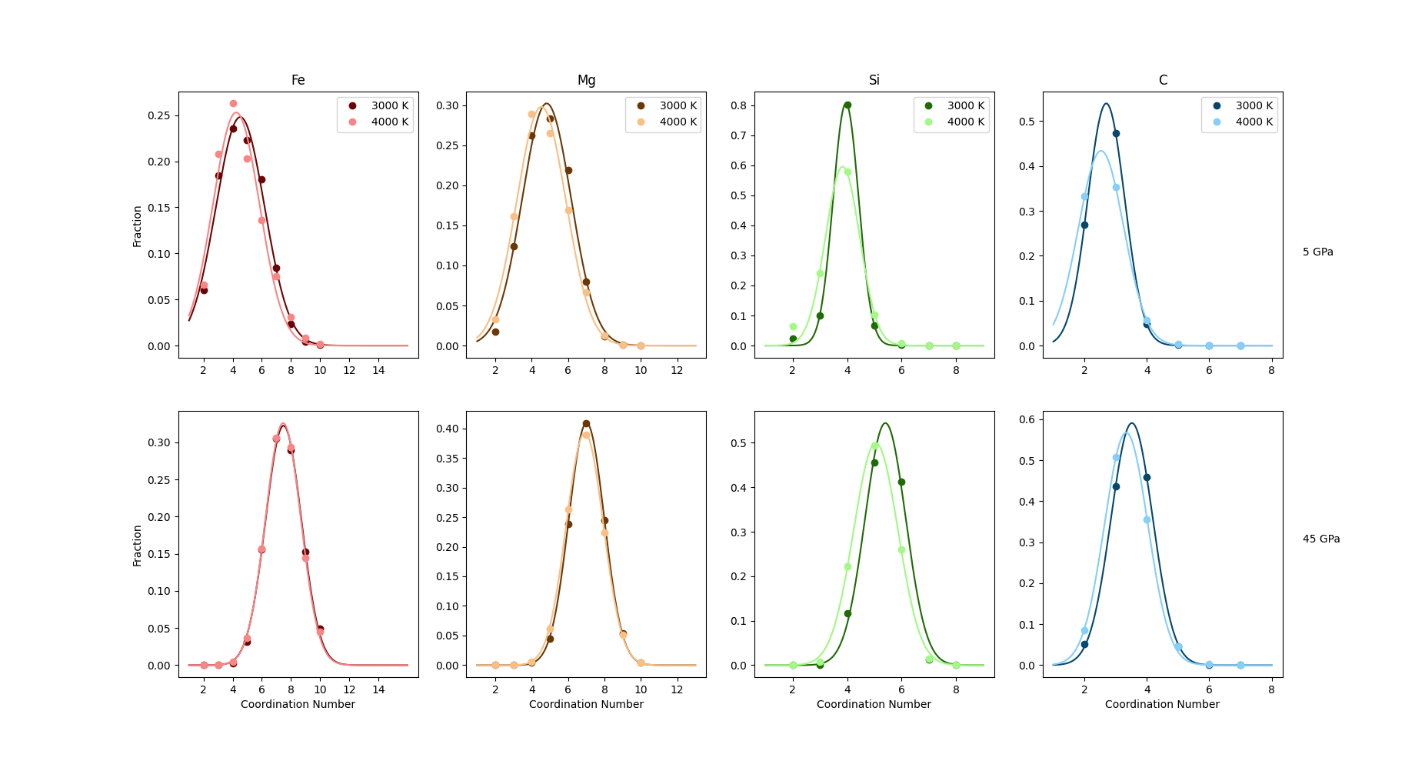


Figure S5. Coordination state distributions as a function of temperature at 5 GPa (top row) and 46 GPa (bottom row). With increasing temperature, distributions broaden and shift to lower average coordination numbers.


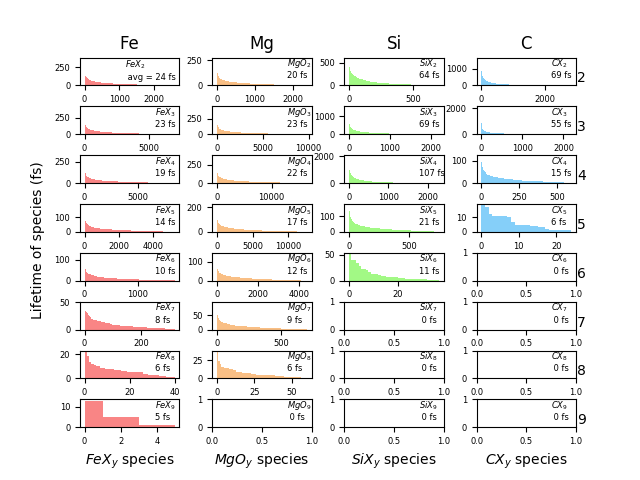


Figure S6. Histogram of coordination state lifetimes for all cations at 4,000 K and 1 GPa. The x axis corresponds to the number of MX_y_ species, and the y axis corresponds to the lifetime of each species. Average coordination lifetimes are displayed in the top right corner of each plot. Coordination states that do not exist are left blank. Lifetime histograms are all skewed with a long tail.


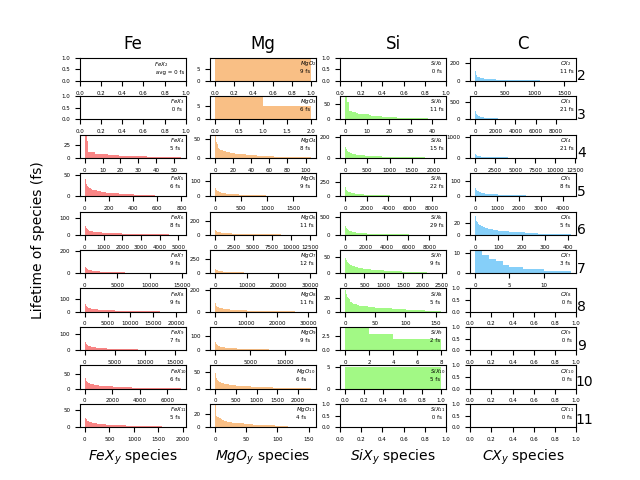


Figure S7. Histogram of coordination state lifetimes for all cations at 4,000 K and 73 GPa. The x axis corresponds to the number of MX_y_ species, and the y axis corresponds to the lifetime of each species. Average coordination lifetimes are displayed in the top right corner of each plot. Coordination states that do not exist are left blank. Lifetime histograms are all skewed with a long tail.


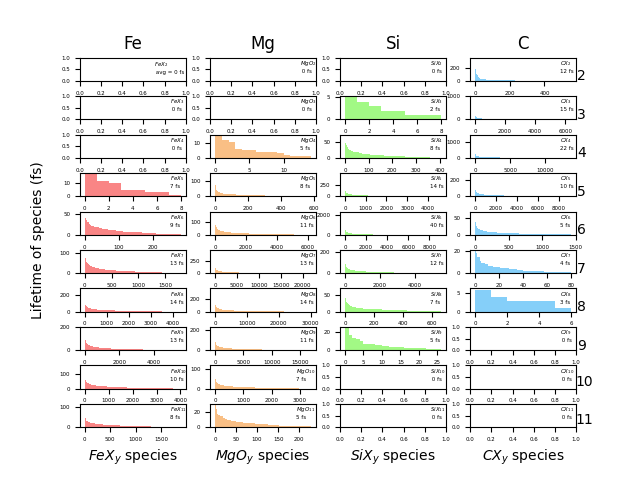


Figure S8. Histogram of coordination state lifetimes for all cations at 4,000 K and 148 GPa. The x axis corresponds to the number of MX_y_ species, and the y axis corresponds to the lifetime of each species. Average coordination lifetimes are displayed in the top right corner of each plot. Coordination states that do not exist are left blank. Lifetime histograms are all skewed with a long tail.


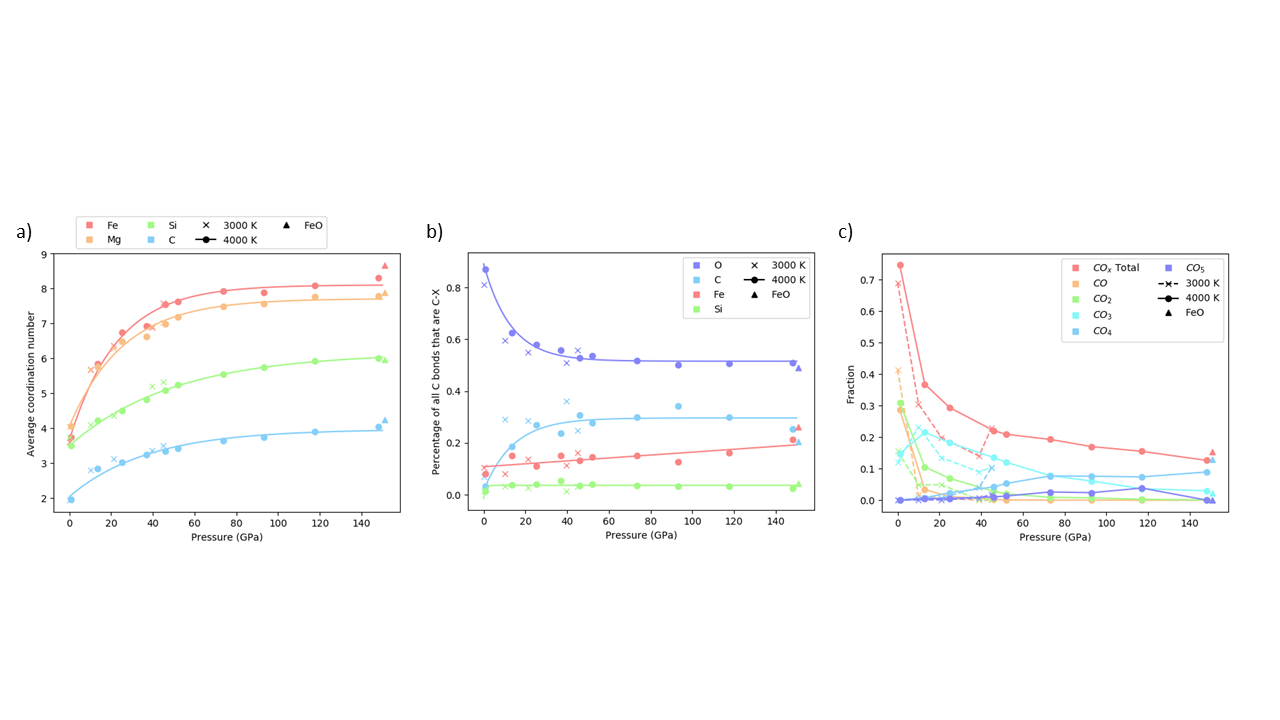


Figure S9. Results of simulations starting with 4 FeO units at 150 GPa and 4,000 K. a) Average cation coordination states as a function of pressure and temperature. Oxidized iron does not significantly change the average coordination state of cations. b) Carbon bond abundances as a function of pressure. The addition of oxidized iron slightly increases the fraction of C-Fe bonds and slightly lowers the fraction of C-C bonds. c) Abundances of carbonate species as a function of pressure and temperature. The addition of oxidized iron slightly increases the abundance of carbonates in the melt.


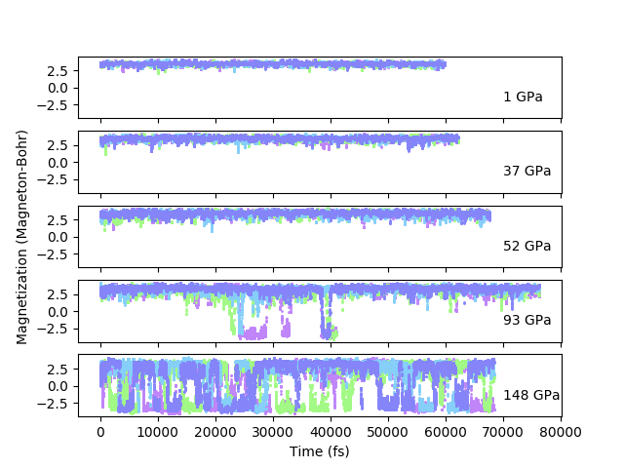


Figure S10. Magnetic moments of four iron atoms versus time across different pressures. With increasing pressure, the local magnetic moments of iron begin to flip, indicating a net loss in magnetism in the melt.
